# Bacterial communities associated with silage of different forage crops in Malaysian climate analysed using 16S amplicon metagenomics

**DOI:** 10.1038/s41598-022-08819-4

**Published:** 2022-05-02

**Authors:** Minhalina Badrul Hisham, Amalia Mohd Hashim, Nursyuhaida Mohd Hanafi, Norafizah Abdul Rahman, Nur Elina Abdul Mutalib, Chun Keat Tan, Muhamad Hazim Nazli, Nur Fatihah Mohd Yusoff

**Affiliations:** 1 Agro-Biotechnology Malaysia Institutes (ABI), National Institutes of Biotechnology Malaysia (NIBM), Ministry of Science, Technology and Innovation (MOSTI), c/o MARDI Headquarters, 43400 Serdang, Selangor Malaysia; 2 Department of Microbiology, Faculty of Biotechnology and Biomolecular Sciences, Universiti Putra Malaysia, 43400 Serdang, Selangor Malaysia; 3 Institutes for Health Systems Research, National Institutes of Health Malaysia (NIH), 40170 Shah Alam, Selangor Malaysia; 4 Department of Crop Science, Faculty of Agriculture, Universiti Putra Malaysia, 43400 Serdang, Selangor Malaysia; 5 Department of Cell and Molecular Biology, Faculty of Biotechnology and Biomolecular Sciences, Universiti Putra Malaysia, 43400 Serdang, Selangor Malaysia; 6 Halal Products Research Institute, Universiti Putra Malaysia, 43400 Serdang, Selangor Malaysia

**Keywords:** Computational biology and bioinformatics, Ecology, Microbiology

## Abstract

Silage produced in tropical countries is prone to spoilage because of high humidity and temperature. Therefore, determining indigenous bacteria as potential inoculants is important to improve silage quality. This study aimed to determine bacterial community and functional changes associated with ensiling using amplicon metagenomics and to predict potential bacterial additives associated with silage quality in the Malaysian climate. Silages of two forage crops (sweet corn and Napier) were prepared, and their fermentation properties and functional bacterial communities were analysed. After ensiling, both silages were predominated by lactic acid bacteria (LAB), and they exhibited good silage quality with significant increment in lactic acid, reductions in pH and water-soluble carbohydrates, low level of acetic acid and the absence of propionic and butyric acid. LAB consortia consisting of homolactic and heterolactic species were proposed to be the potential bacterial additives for sweet corn and Napier silage fermentation. Tax4fun functional prediction revealed metabolic pathways related to fermentation activities (bacterial division, carbohydrate transport and catabolism, and secondary metabolite production) were enriched in ensiled crops (*p* < 0.05). These results might suggest active transport and metabolism of plant carbohydrates into a usable form to sustain bacterial reproduction during silage fermentation, yielding metabolic products such as lactic acid. This research has provided a comprehensive understanding of bacterial communities before and after ensiling, which can be useful for desirable silage fermentation in Malaysia.

## Introduction

Animal feed plays a pivotal role in ensuring the healthy growth of domesticated livestock in husbandry, which takes almost 70% of the animal rearing cost. The increasing cost of animal feed importation in Malaysia has caused farmers to downsize their local livestock farming, leading to insufficient local beef production^1^. Self-sufficiency of beef in Malaysia is only 23.7% in 2020^2^. As the Malaysian government has imposed a ceiling price for livestock produce, self-production of local feed with comparable quality and quantity to imported feed has become necessary to keep the meat price low for the local market. Therefore, silage production has become an alternative to ensure that local meat can be marketed below the stipulated price limit. Silage is not commonly utilised in Malaysia unlike in Europe, America and China, as Malaysia has a tropical climate that permits continuous farming of fresh crops throughout the year. However, farmers may consider replacing the expensive imported feed with silage for cheaper livestock production. Corn and Napier are the popular selection of forage in Malaysia. The locally grown corn forage can be used to make high-quality feed and silage because of its high nutritive quality compared to other crops such as legumes, alfalfa and soybean. Napier grass is the popular cultivated feed for ruminants^3^ in Malaysia, and it has high yield production with greater dry matter (DM) than other tropical grasses such as Rhodes grass and Guinea grass^4^.

Silage is prepared through ensiling of selected crops. Ensiling is a complex process as it involves vast microbial communities whereby the epiphytic microbial communities of fresh forages play a critical role in ensiling. Microbial fermentation produces an array of metabolites, which can change nutritive aspects of forage^5^. By ensiling crops, the energy content of the crops is preserved, resulting good nutritional value to be given as feed to ruminants. Preservation of the nutritional value of the fresh forage can be achieved by lowering the pH and maintaining anaerobic conditions for the survival of LAB. LAB play an essential role in ensiling by converting fermentable carbohydrates into organic acids. During carbohydrate fermentation, LAB will produce lactic acid (LA) and reduce the pH value, which can inhibit the spoilage microorganisms and stabilise the silage. Despite the active effort to produce silage in Malaysia, silage produced in tropical countries such as Malaysia has poor silage quality, which easily deteriorates or often encounters spoilage^6^.

Under hot or tropical conditions, silages are more prone to aerobic spoilage^6^ as high temperature is favourable for the growth of spoilage microorganisms and insufficient production of antifungal compounds to inhibit their growth^7^. In addition, acetic acid is predominant as a fermentation product due to heterofermentative ensiling in tropical regions^8,9^. According to Li and Nishino, silage preparation at high moisture and prolonged ensiling can also escalate the acetic acid fermentation. Corn silage prepared in tropical region usually has lower digestible energy content for ruminate^6^ compared with that prepared in temperate climates. Silage quality can be determined by measuring its physicochemical properties that indicate successful silage fermentation such as DM, pH, ethanol concentration and organic acid (LA, butyric acid, propionic acid and acetic acid) content. Good silage quality is often shown by a decrease in water-soluble carbohydrates (WSC) and pH, increase in LA content and low amount of acetic acid, butyric acid and propionic acid^5^.

In Malaysia, studies on bacterial communities involved in ensiling of corn and Napier silage are limited. The common LAB genera involved in ensiling include *Lactobacillus*, *Pediococcus*, *Lactococcus*, *Enterococcus*, *Streptococcus* and *Leuconostoc*^10^. Some of these genera are abundant in tropical grass silages^11^. In general, silage fermentation in temperate regions is conducted in silo drum or bunker silo, in addition to the use of bacterial additive to improve silage quality^11–15^. The use of bacterial additive can improve silage nutritive quality and practices since 1990s^10^, where it decreases DM losses and enhances aerobic stability. The most common bacterial additive used to improve silage fermentation is LAB^16^. The addition of LAB as an inoculum ensures sufficient abundance of LAB present to kick start fermentation, thereby preventing undesirable microorganisms from predominating. Thus, extensive attempts to profile bacterial communities involved in silage fermentation are necessary to reveal important taxa that contribute towards enhanced silage quality in tropical countries.

We hypothesised that the bacterial community of fresh forage and ensiled crops will change significantly after ensiling, and different crops will exhibit different bacterial community profiles. This study aimed to determine bacterial community changes associated with silage fermentation using amplicon metagenomics in two different forage crops commonly used in Malaysia, namely, Napier and sweet corn. This study has also identified endogenous LAB species associated with Napier and corn silage, which are potentially useful as bacterial additives to improve silage fermentation and quality in Malaysia.

## Results

### Silage fermentation characteristics indicate good silage quality

Table 1 shows fermentation properties of sweet corn and Napier. DM and WSC for sweet corn decreased significantly (*p*-value = 0.00610 and *p*-value = 0.04993, respectively) after 21 days of ensiling. The pH observed in fresh sweet corn and Napier was 4.00 and 4.24, respectively. The pH of sweet corn and Napier silage significantly reduced below 4 (*p*-value = 0.00978 and *p-*value = 0.00001, respectively) after 21 days. Napier silage showed the lowest pH at 3.37. Crude protein (CP) in Napier decreased (*p*-value = 0.00257), whereas that in sweet corn increased (*p-*value = 0.00001) after ensiling. The colony-forming unit (CFU) count of LAB on *de Man*, Rogosa, Sharpe agar (MRS) and LA content in both forage crops significantly increased (*p*-value = 0.00001 and *p*-value = 0.00048, respectively). LA concentration in Napier silage was significantly higher than that in sweet corn silage (*p-*value = 0.00800). Butyric acid and propionic acid were not detected in silage, whereas the concentration of acetic acid in both silages was below 1% DM.

**Table 1:** Fermentation characteristics of sweet corn and Napier before and after ensiling for 21 days.

Sample	S0	S21	*p*-value	N0	N21	*p*-value
DM (%)	23.54 ± 1.655^a^	16.98 ± 0.296^b^	0.00610	14.66 ± 0.364^a^	17.37 ± 0.767^a^	0.33424
pH	4.00 ± 0.083^d^	3.74 ± 0.010^bc^	0.00978	4.24 ± 0.050^e^	3.37 ± 0.013^a^	0.00001
WSC (%DM)	17.32 ± 5.072^d^	8.23 ± 2.380^ab^	0.04993	24.45 ± 4.075^ab^	7.45 ± 4.318^a^	0.97777
LA (%DM)	0.287 ± 0.079^a^	2.210 ± 0.321^b^	0.00001	0.713 ± 0.135^a^	3.060 ± 0.487^c^	0.00001
AA (%DM)	nd	0.740 ± 0.062^a^		nd	0.910 ± 0.125^a^	
BA (%DM)	nd	ND	nd	nd	ND	nd
PA (%DM)	nd	ND	nd	nd	ND	nd
CP(%DM)	2.71 ± 0.146^ab^	5.62 ± 0.150^c^	0.000001	3.37 ± 0.318^a^	2.00 ± 0.187^b^	0.00257
Viable aerobic bacteria (log cfu/g FM)	6.09 ± 0.109^a^	6.00 ± 0.000^a^	1.00000	6.38 ± 0.175^a^	6.81 ± 0.562^a^	0.99274
Viable lactic acid bacteria (log cfu/g FM)	3.04 ± 0.681^a^	6.78 ± 0.042^b^	0.00048	3.56 ± 0.610^a^	7.42 ± 0.331^b^	0.00048

*S0* fresh sweet corn, *S21* sweet corn silage, *N0* fresh Napier, *N21* Napier silage, *DM* dry matter, *WSC* water-soluble carbohydrates, *LA* lactic acid, *AA* acetic acid, *BA* butyric acid, *PA* propionic acid, *CP* crude protein, *FM* fresh materials, *nd* not determined, *ND* not detected.

Values in the same row followed by different letters are significantly different (*p*-value < 0.05).

### Bacterial communities shifted to fermentative and anaerobic bacteria after ensiling

**Figure 1:**
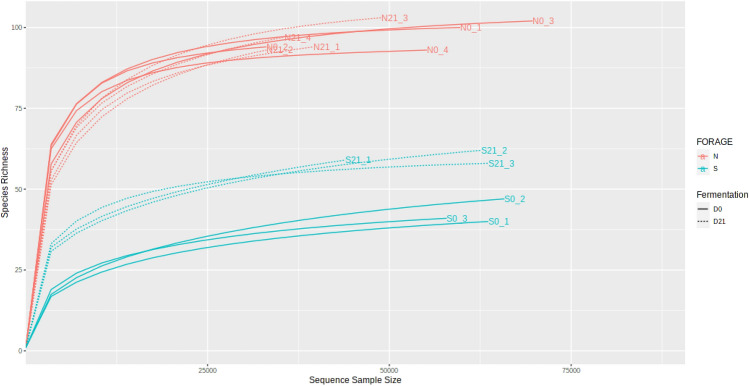
Rarefaction curve of the observed operational taxonomic units (OTUs) of sweet corn and Napier for fresh forage and silage using unrarefied sequences (S0, Fresh sweet corn; S21, Sweet corn silage; N0, Fresh Napier; N21, Napier silage).

**Figure 2:**
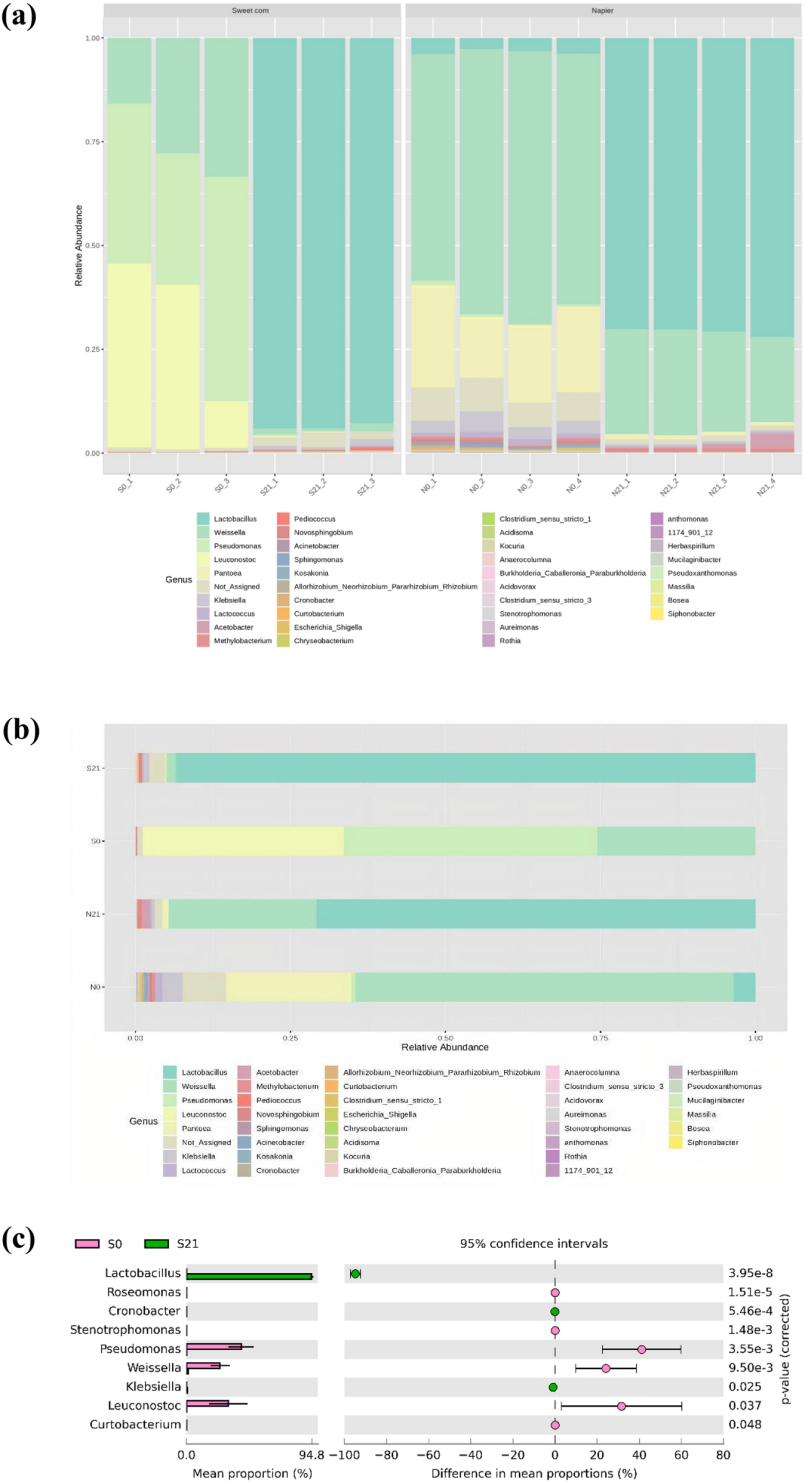

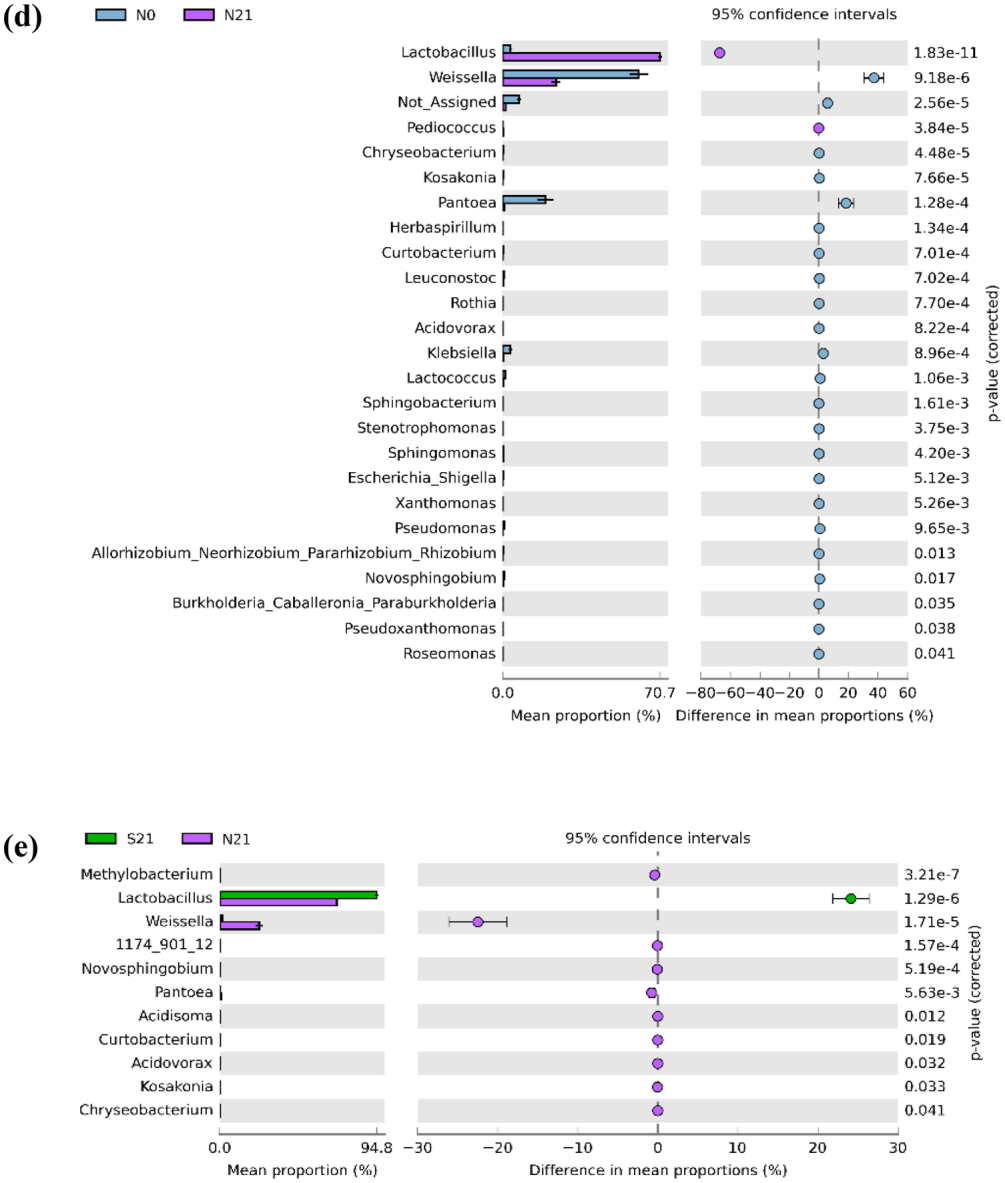
Composition of bacterial communities before and after ensiling using unrarefied sequences at genus level: **(a)** each sample, **(b)** group sample, **(c)** extended error bar comparing fresh sweet corn (S0) and sweet corn silage (S21), **(d)** extended error bar comparing fresh Napier (N0) and Napier silage (N21) and **(e)** extended error bar comparing S21 and N21 using t-test at *p-*value < 0.05.

Total read counts of 14 samples obtained by 16S rRNA gene amplicon sequencing (V3–V4 regions) were 742,298, which were clustered into 408 operational taxonomic units (OTUs) at 97% sequence similarity. The sequencing results are summarised in Supplementary Table S1. The rarefaction curve showed that all samples reached plateau. The triplicate silages showed similar rarefaction curves, which were grouped in accordance with their respective crops, and Napier showed higher species richness than sweet corn silage (Fig. 1). The percentage abundance of bacterial genera is shown in Supplementary Table S2. The major bacterial genera in fresh sweet corn were *Pseudomonas* (40.89%), *Leuconostoc* (32.49%) and *Weissella* (25.43%), whereas Napier was dominated by *Weissella* sp. (61.00%) and *Pantoea* (19.65%) (Fig. 2, Supplementary Table S2). However, the presence of these genera decreased after ensiling (Fig. 2c,d). In fresh materials, the abundance of *Lactobacillus* sp. was only 0.04% in sweet corn and 3.50% in Napier. After ensiling, homofermentative and heterofermentative *Lactobacillus* sp. increased remarkably by 93.78% and 70.78% in sweet corn and Napier silages, respectively. When compared between the two silages, *Lactobacillus* was the signature of sweet corn silage, whereas *Weissella*, an obligate heterofermentative bacteria, characterised the Napier silage based on their differential abundance (Fig. 2e). High proportion of homofermentative and heterofermentative species, namely, *Lactobacillus brevis* and *Lactobacillus casei* in sweet corn silage, and *Lactobacillus fermentum* in Napier silage were also observed upon ensiling (Supplementary Fig. S1).

**Figure 3:**
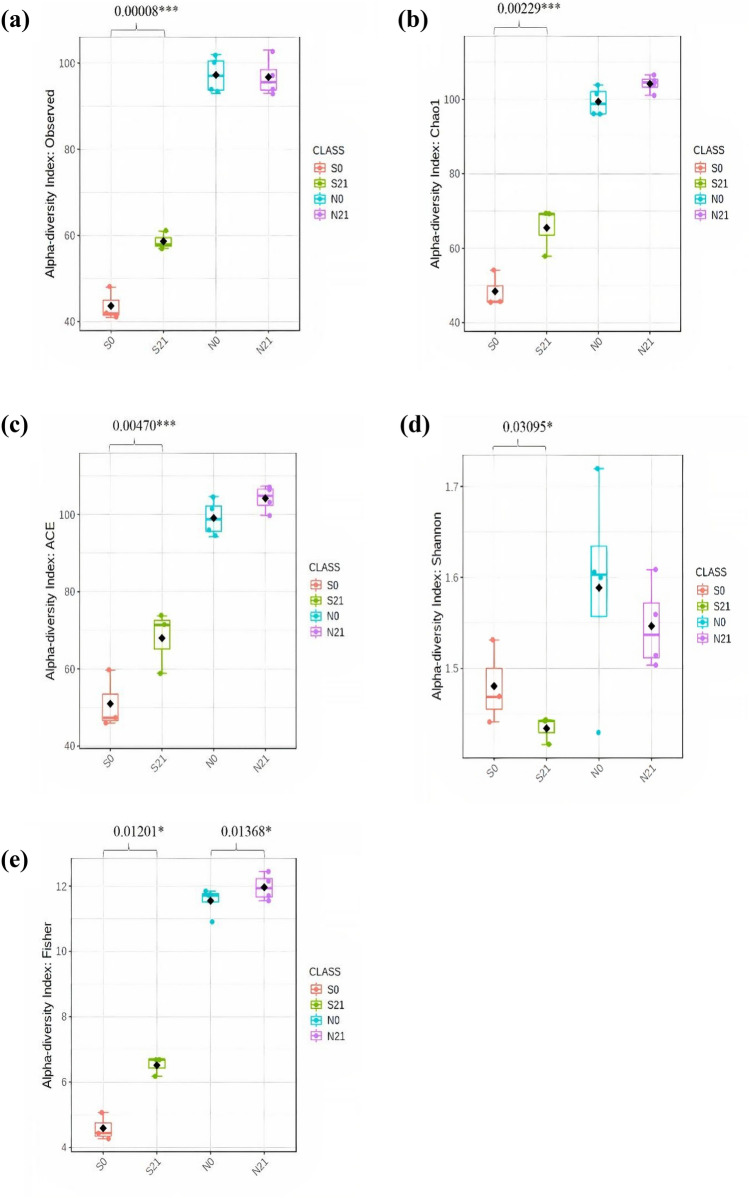
Alpha diversity of bacterial communities before and after ensiling for sweet corn and Napier: **(a)** observed OTU (F-value: 162.3; *p-*value = 0.00001), **(b)** Chao1 (F-value: 125.38; *p-*value = 0.00001), **(c)** ACE (F-value: 64.905; *p-*value = 0.00001), **(d)** Fisher (F-value: 291.78; *p-*value = 0.00001), **(e)** Shannon (F-value: 1.4881; *p-*value = 0.27669) indices measured at feature level (S0, Fresh sweet corn; S21, Sweet corn silage; N0, Fresh Napier; N21, Napier silage).

**Figure 4:**
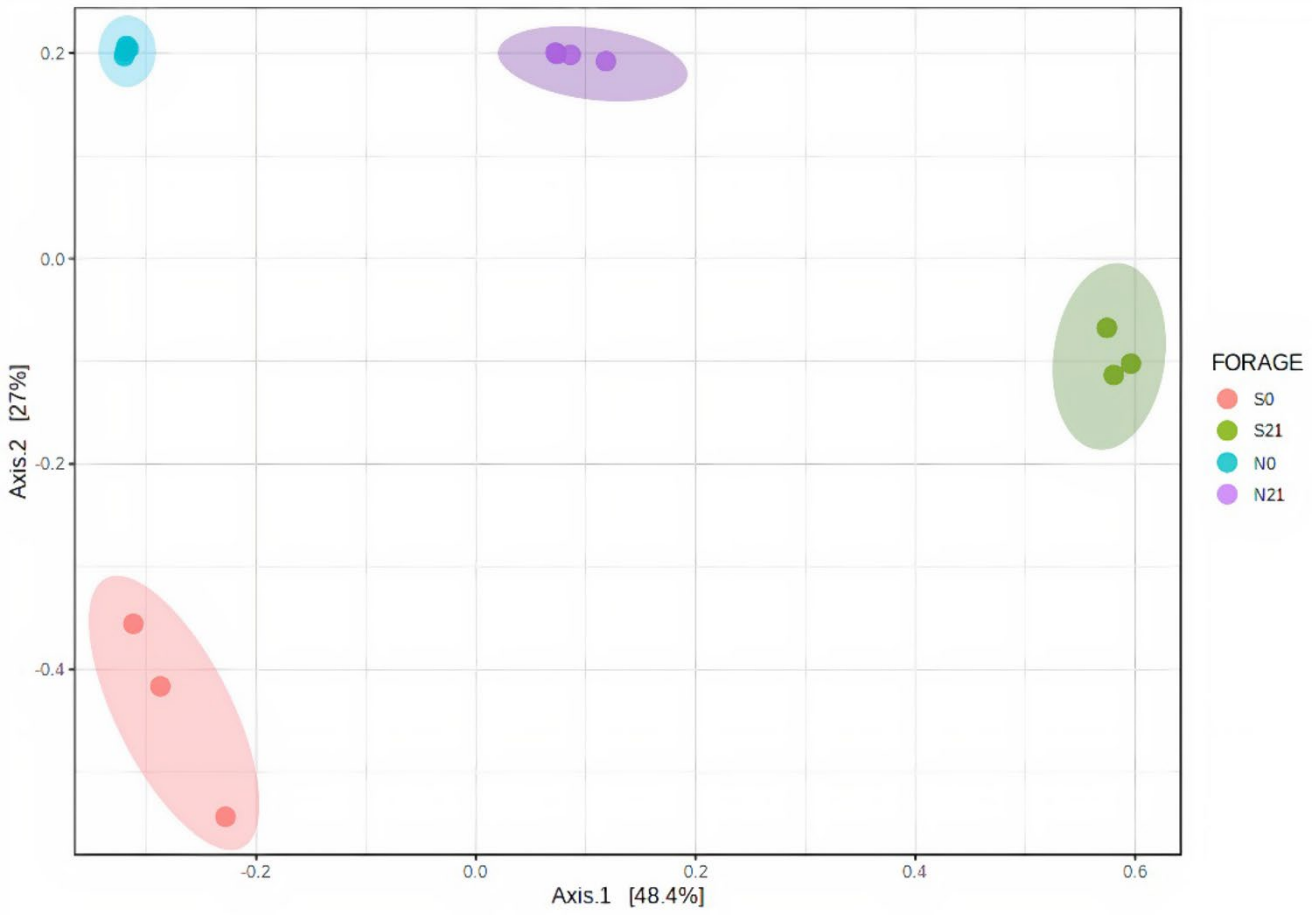
Principal coordinate analysis (PCoA) derived from the Bray–Curtis distance between fresh forage and silage of sweet corn and Napier. Coloured dots represent different forage crops. PERMANOVA value, F-value: 107.71, R-squared: 0.96998, *p-*value < 0.001 (S0, Fresh sweet corn; S21, Sweet corn silage; N0, Fresh Napier; N21, Napier silage).

Alpha diversity was plotted across samples and visualised as boxplots for each group sample at feature level (Fig. 3) to assess the diversity of bacterial communities after ensiling. After 21 days of fermentation, the observed OTU, Chao1, ACE and Fisher indices in sweet corn increased (*p-*value = 0.00008, *p-*value = 0.00229, *p-*value = 0.00470, *p-*value = 0.01201, respectively), whereas Shannon index decreased (*p*-value = 0.03095). A similar pattern was observed in Napier despite the statistically insignificant increments except for the Fisher index. The species richness and diversity of fresh and Napier silage were greater than that of sweet corn. The observed OTU and Chao 1 indices in sweet corn silage increased after fermentation. This result was in agreement with the Venn diagram (Supplementary Fig. S3), where the number of OTUs of ensiled sweet corn was higher than that of fresh sweet corn. As shown in the Venn diagram (Supplementary Fig. S3), 62 OTUs were shared between fresh and ensiled sweet corn, whereas 148 OTUs were shared between fresh and ensiled Napier. The number of unique OTUs in sweet corn silage was higher than that in fresh sweet corn, but opposite result was found in Napier. Two-dimensional principal coordinate analysis (PCoA) was plotted using the Bray–Curtis distance matrix (Fig. 4). Bacterial communities of fresh forage and silage samples were distinctly separated before and after ensiling (F-value: 107.71, R-squared: 0.96998, *p-*value < 0.001).

**Figure 5:**
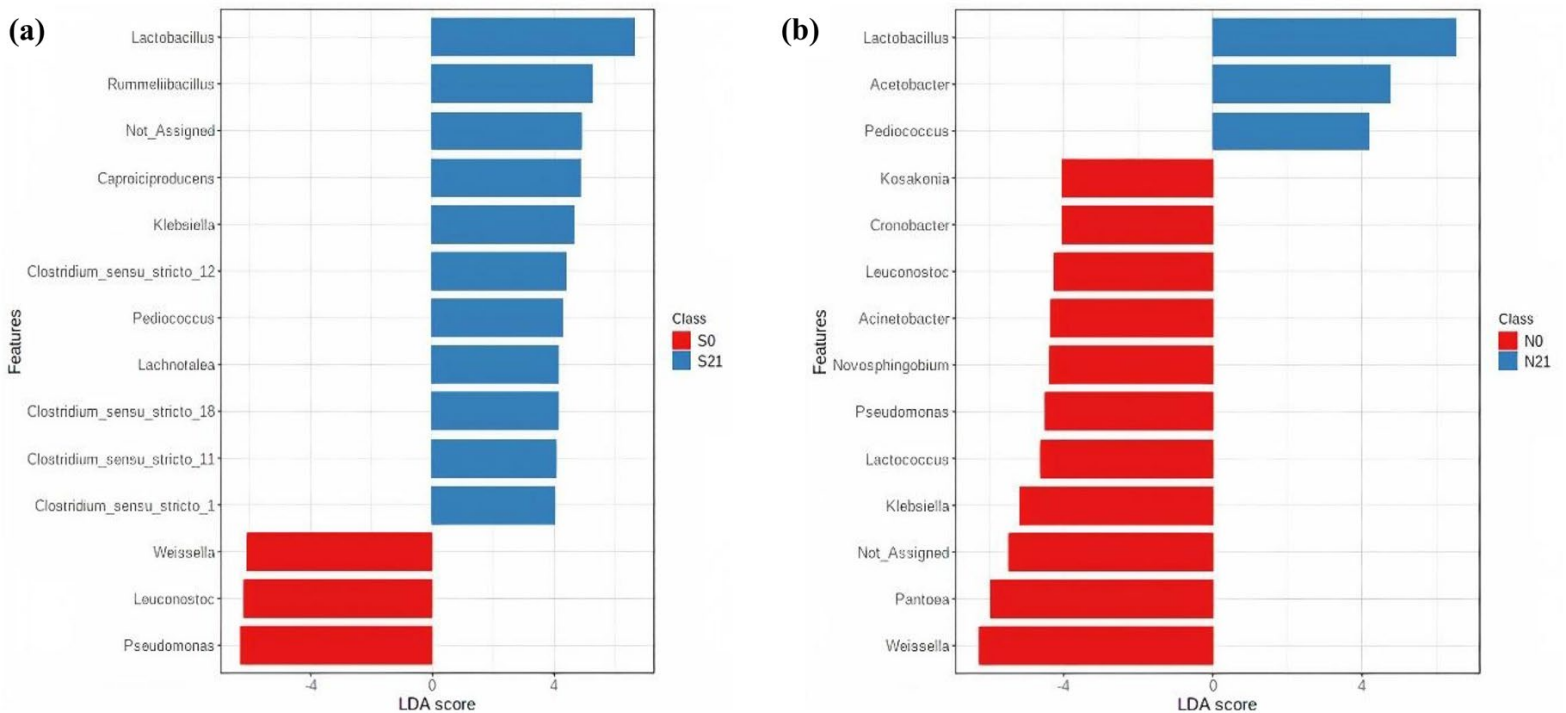
Graphics of linear discriminant analysis (LDA) effect size (LEfSe) between fresh forage and silage, **(a)** sweet corn and **(b)** Napier, at genus level. Red indicates fresh forage and blue for silage. The threshold on the logarithmic LDA score for discriminative features was set to 4.0 at *p*-value < 0.05 (original *p*-value for sweet corn and FDR-adjusted for Napier) (S0, Fresh sweet corn; S21, Sweet corn silage; N0, Fresh Napier; N21, Napier silage).

**Figure 6:**
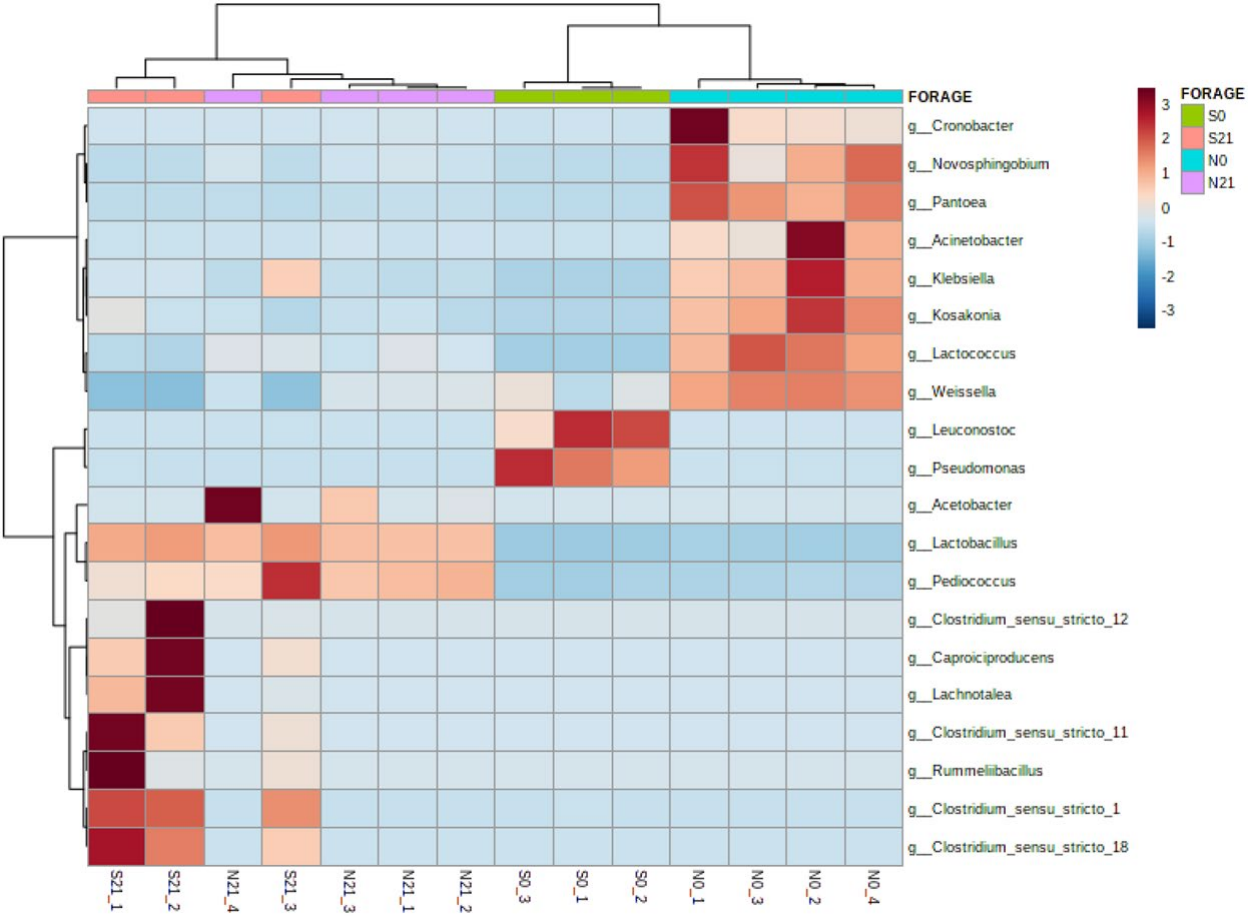
Heatmap constructed using differentially abundant taxa from LEFSe analysis at the genus level. Pearson distance was used with Ward clustering algorithm by clustering samples based on the current clustering algorithm. Red and blue indicate a higher and lower abundance, respectively (S0, Fresh sweet corn; S21, Sweet corn silage; N0, Fresh Napier; N21, Napier silage).

Linear discriminant analysis effect size (LEfSe) analysis was conducted at the genera level with linear discriminant analysis (LDA) score > 4.0 capped at *p*-value < 0.05 for Napier and sweet corn. Fourteen differentially abundant genera each in sweet corn and Napier were identified. Based on LEfSe (Fig. 5), the common biomarker taxa of fresh sweet corn and fresh Napier were *Pseudomonas*, *Leuconostoc* and *Weissella*. The discriminatory genera for sweet corn silage were *Lactobacillus*, *Rummeliibacillus*, *Caproiciproducens*, *Klebsiella*, *Pediococcus*, *Clostridium* and *Lachnotalea* (Fig. 5a). By contrast, in Napier silage (Fig. 5b), *Lactobacillus*, *Acetobacter* and *Pediococcus* were discriminant. Heatmap was then constructed (Fig. 6) based on the genera observed in LEfSe analysis. LEfSe analysis at feature level (Supplementary Fig. S4) and heatmap based on discriminatory OTUs (Supplementary Fig. S5) were also included. The clusters were separated by forage type and fermentation status.

### Enriched homofermentative and heterofermentative LAB after ensiling were positively correlated

**Figure 7:**
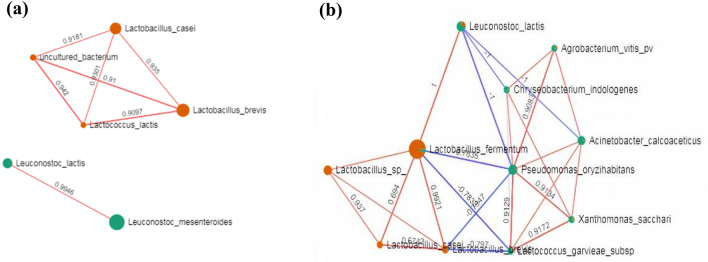
Taxa–taxa interaction at species level using SPARCC set at permutation value of 200, *p-*value < 0.05, correlation > 0.6: **(a)** sweet corn and **(b)** Napier. Blue edges represent negative correlation, whereas orange edges represent positive correlation. The proportion of bacterial taxa is shown in green for fresh crops and orange for silage.

Co-occurrence network analyses were conducted to evaluate the bacterial interactions in fresh forage crops and silages. The correlations were calculated using SparCC analysis with correlation value of > 0.66 with 200 permutations at *p*-value < 0.05 (Fig. 7). The SPARCC correlation amongst selected species is shown in Supplementary Tables S3 and S4. The taxa network in sweet corn (Fig. 7a) consists of six nodes with seven positive correlations. *Leuconostoc lactis* and *Leuconostoc mesenteroides* that were predominant in sweet corn before ensiling showed a strong positive correlation (*r* = 0.9946, *p*-value = 0.02490). A trio of LAB, that is, homofermentative *Lactococcus lactis* and *Lactobacillus casei*, and heterofermentative *Lactobacillus brevis* were positively correlated with each other in sweet corn silage (*Lactococcus lactis* and *Lactobacillus brevis*, *r* = 0.9097, *p-*value = 0.03980; *Lactococcus lactis* and *Lactobacillus casei*, *r* = 0.9301, *p*-value = 0.03980; *Lactobacillus casei* and *Lactobacillus brevis*, *r* = 0.9350, *p*-value = 0.04480). For Napier (Fig. 7b), 11 nodes with seven negative correlations and 18 positive correlations were identified. Another trio of homofermentative *Lactobacillus casei*, and heterofermentative *Lactobacillus fermentum* and *Lactobacillus brevis* were enriched with positive correlation with one another (*Lactobacillus casei* and *Lactobacillus brevis*, *r* = 0.6742, *p*-value = 0.01000; *Lactobacillus casei* and *Lactobacillus fermentum*, *r* = 0.6940, *p*-value = 0.04980; *Lactobacillus brevis* and *Lactobacillus fermentum*, *r* = 0.9921, *p*-value = 0.01000). *Leuconostoc lactis* showed strong negative correlation with *Pseudomonas oryzihabitans* (*r* =  − 1, *p*-value = 0.04480) and *Acinetobacter oryzihabitans* (*r* =  − 1, *p*-value = 0.04980). It also has a strong positive correlation with *Lactobacillus fermentum* (*r* = 1, *p*-value = 0.04480). In the Napier sample, *Lactococcus garvieae* subsp *garvieae* was positively correlated with the bacterial genera that have been previously reported as plant pathogens such as *Xanthomonas sacchari*, *Pseudomonas oryzihabitans* and *Acinetobacter calcoaceticus*, but this result was not observed in sweet corn.

### LAB were enriched along with low pH and high LA content after fermentation

**Figure 8:**
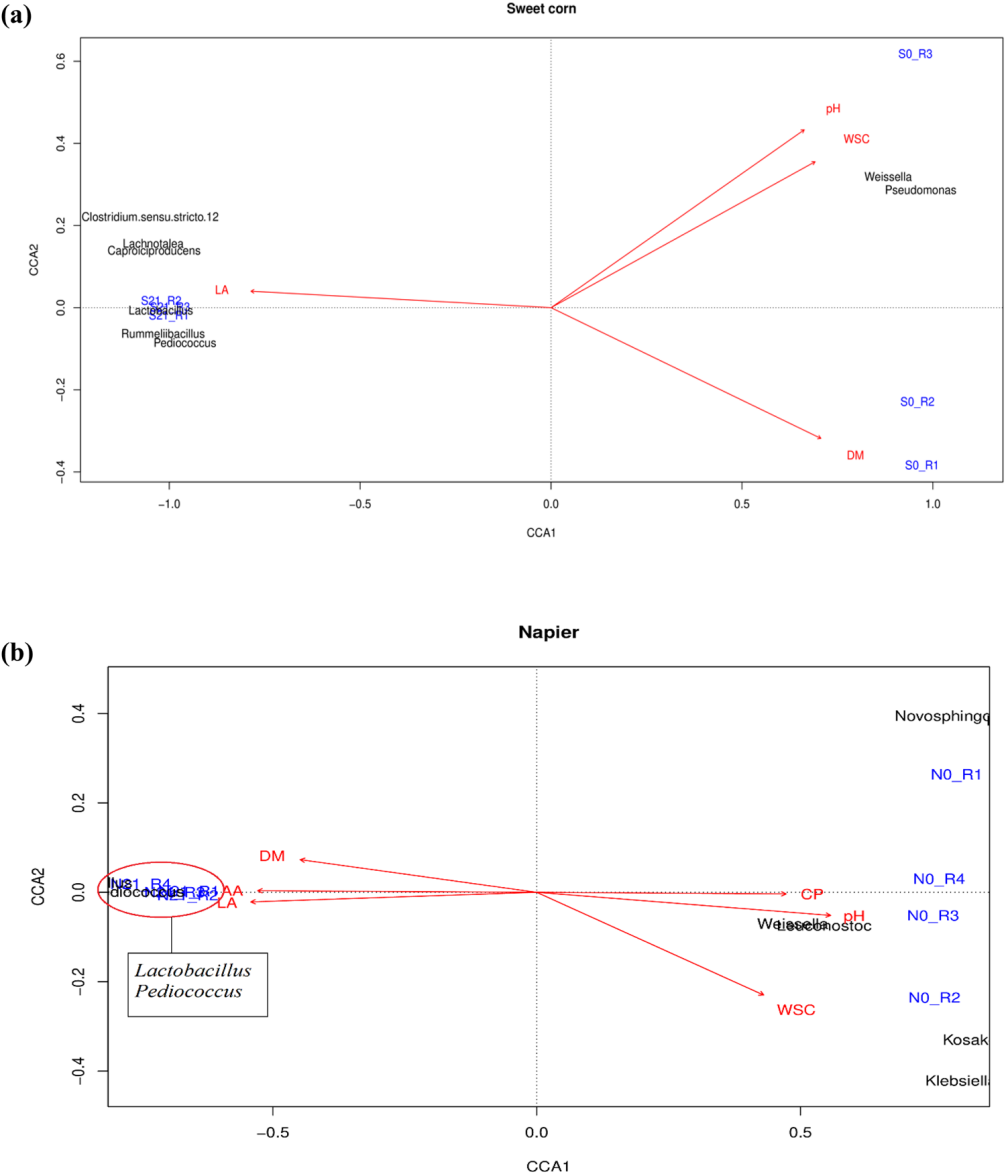
Correlation between physicochemical properties and bacterial taxa of fresh forage crops and silage samples by CCA analysis: **(a)** sweet corn (total variance explained: 98.1%, *p* < 0.05) and **(b)** Napier (total variance explained: 99.8%, *p* < 0.05).

CCA analysis of significant physicochemical parameters and differentially abundant taxa was constructed on the basis of each forage crop. In fresh sweet corn (Fig. 8a), *Weissella* and *Pseudomonas* were abundant at high pH and WSC. After ensiling, *Lactobacillus*, *Rummeliibacillus*, *Clostridium*, *Lachnotalea*, *Pediococcus* and *Caproiciproducens* were predominant in sweet corn silage along with increased LA content but low DM, WSC and pH. On the contrary, for fresh Napier (Fig. 8b), *Leuconostoc* and *Weissella* were abundant at high CP, WSC and pH levels but low DM, acetic acid and LA content. After 21 days of fermentation, *Lactobacillus* and *Pediococcus* were highly abundant in Napier silage. These genera showed association with low WSC, CP and pH but high LA and acetic acid content. Analysis at the feature level also showed that several OTUs belonging to LAB were positively correlated with the high LA content of ensiled crops (Supplementary Fig. S6).

### Enrichment of metabolic functions associated with active bacterial reproduction, carbohydrate transport and turnover and secondary metabolite production after ensiling

The prediction of metabolic functions during crop ensiling provides a comprehensive understanding of the process to produce high-quality silage particularly in a tropical country where silage is easily spoiled and produced at low quality. The bacterial community OTUs were used to predict Kyoto Encyclopedia of Genes and Genomes (KEGG)^17–19^ functional profiles by analysing the OTUs against the KEGG database using Tax4Fun in MicrobiomeAnalyst. In total, 125 metabolic pathways were predicted.

**Figure 9:**
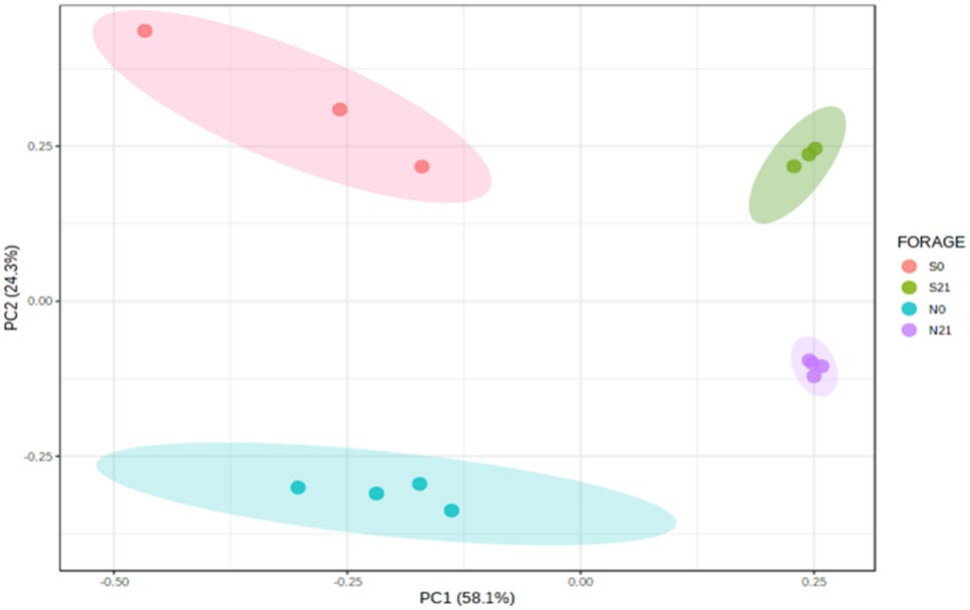
Principal coordinate analysis (PCoA), calculated using the Euclidean distance algorithm between fresh crops and silage for sweet corn and Napier based on KEGG functional profiles predicted by Tax4Fun. Coloured dots represent different forage crops (S0, Fresh sweet corn; S21, Sweet corn silage; N0, Fresh Napier; N21, Napier silage).

**Figure 10:**
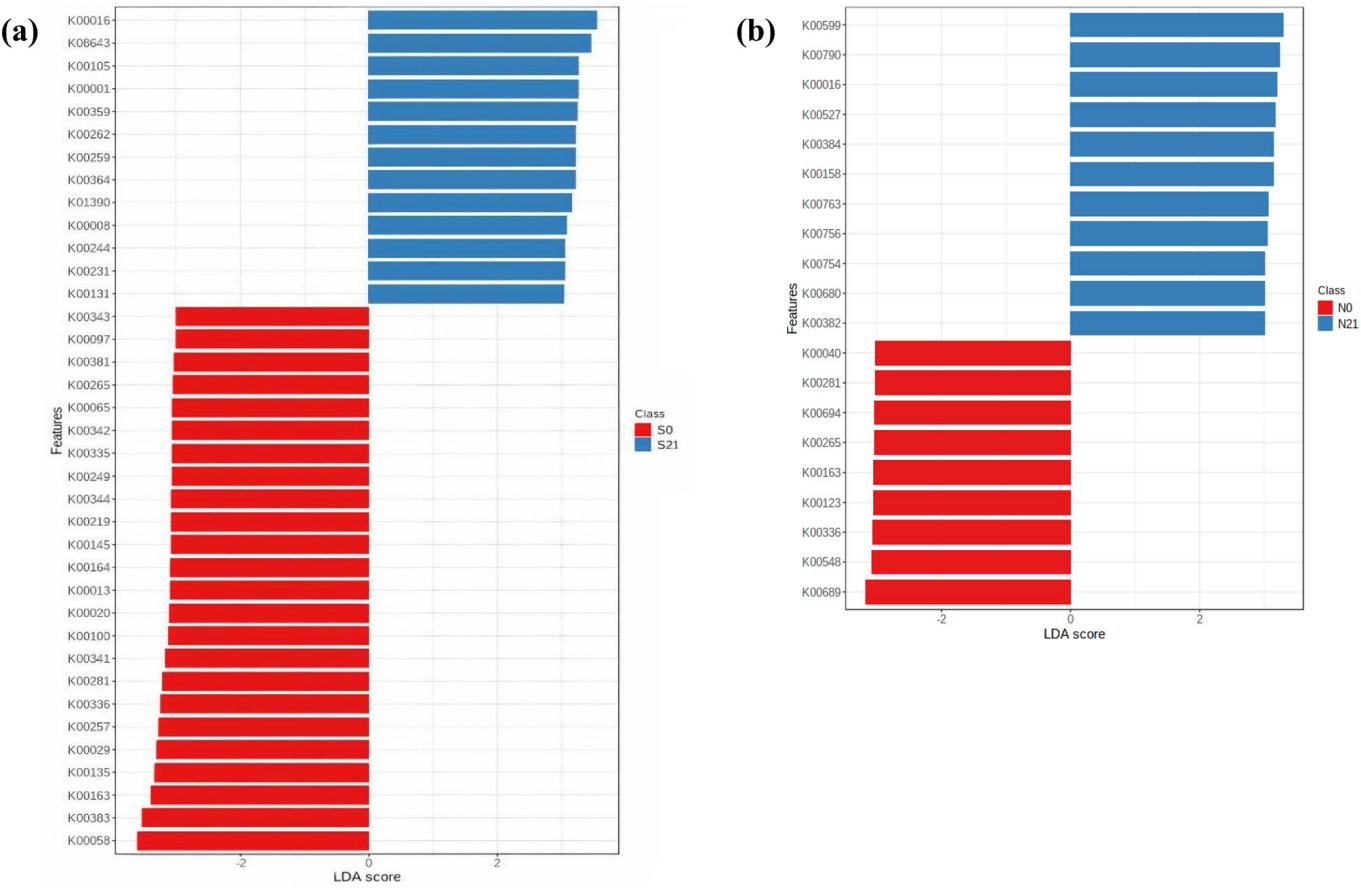
Graphics of linear discriminant analysis (LDA) effect size (LEfSe) of the functional prediction profiles using Tax4Fun between fresh forage and silage. The threshold on the logarithmic LDA score for discriminative features was set to 3.0 at FDR-adjusted *p*-value < 0.05 (S0, Fresh sweet corn; S21, Sweet corn silage; N0, Fresh Napier; N21, Napier silage).

**Figure 11:**
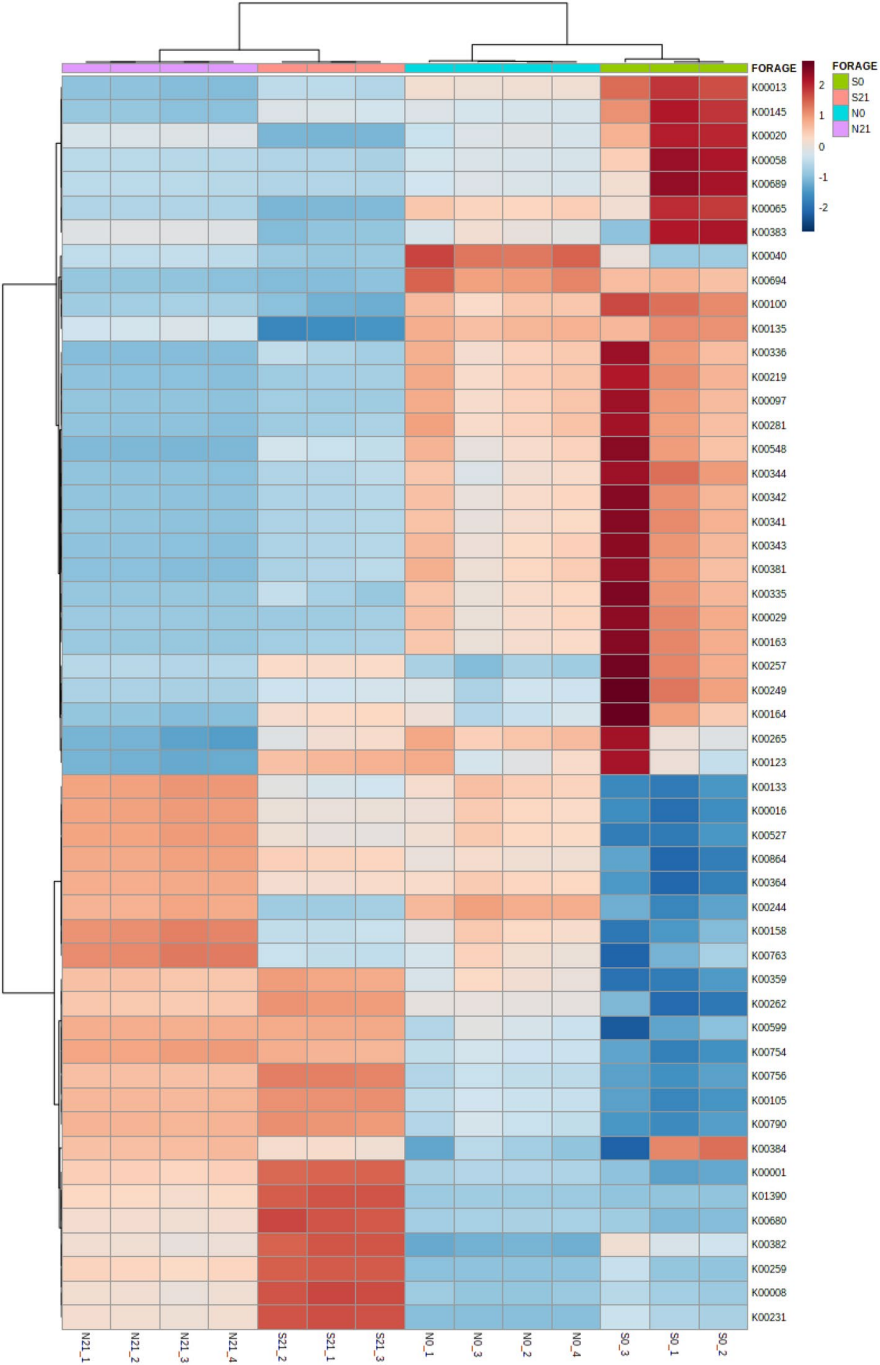
Heatmap constructed using differentially abundant KEGG functional profiles  using Tax4Fun based on LEFSe analysis shown in Fig. 10. Pearson distance was used with the Ward clustering algorithm based on the current clustering algorithm. Red and blue indicate a higher and lower abundance, respectively.

**Figure 12:**
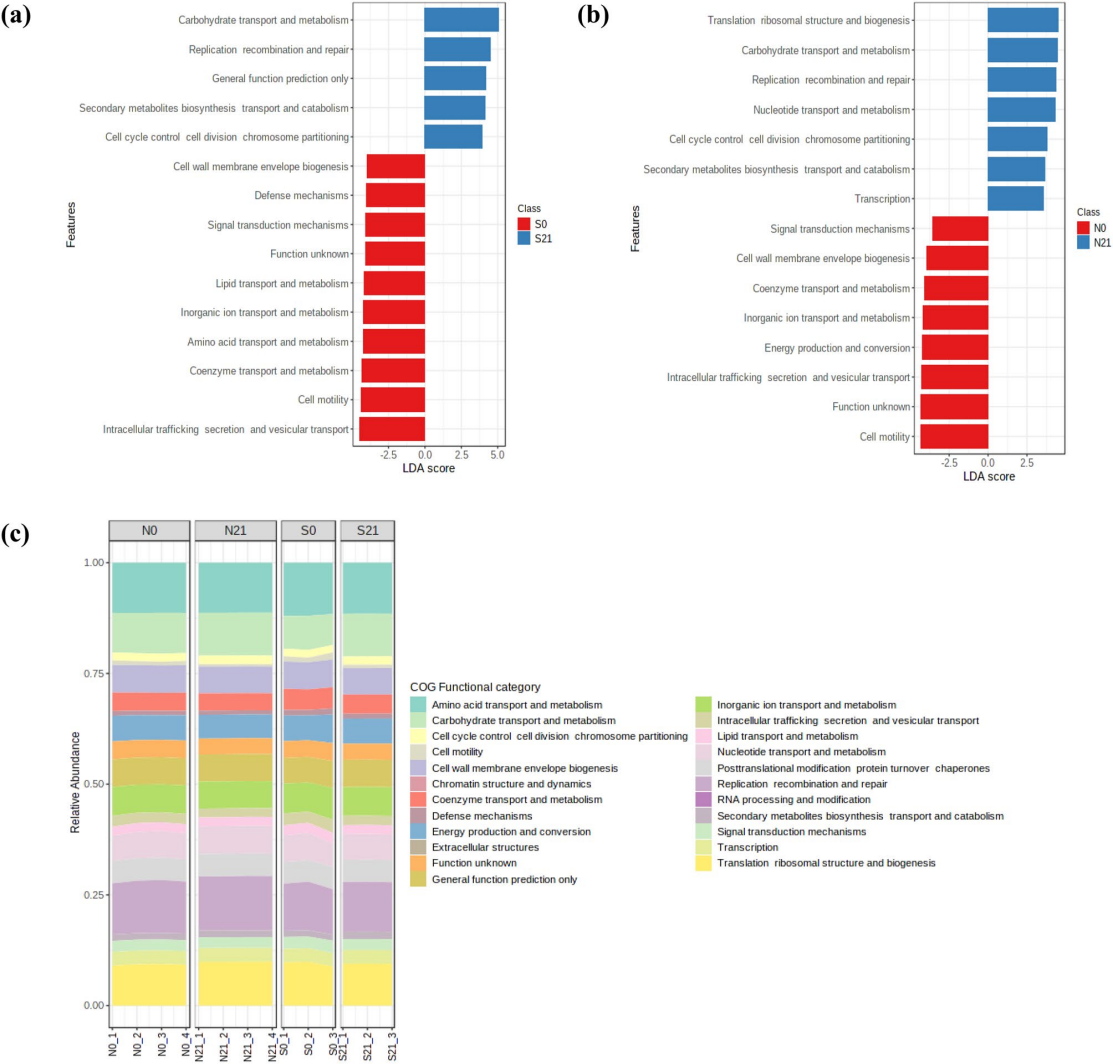
Stacked bar chart and graphic of linear discriminant analysis (LDA) effect size (LEfSe) of COG functional categories across samples, (**a**) sweet corn and (**b**) Napier (**c**) relative abundance of COG functional categories across samples (S0, Fresh sweet corn; S21, Sweet corn silage; N0, Fresh Napier; N21, Napier silage).

The beta diversity (Fig. 9) of the predicted functional profiles shows that the KEGG pathways of fresh forage and silage for both crops were distinctly separated. The LDA score of the functional prediction profile was set at 3.0 (FDR-adjusted *p*-value < 0.05, Fig. 10). In the sweet corn sample, 37 discriminatory KEGG functional orthologs (KOs) were identified, 13 of which were differentially abundant in fresh sweet corn. Twenty KOs were detected in the Napier sample. The definition of each KO was tabulated in Supplementary Table S5. K00265 (glutamate synthase [NADPH] large chain), K00163 (pyruvate dehydrogenase E1 component), K00336 (NADH-quinone oxidoreductase subunit G) and K00281 (glycine dehydrogenase) were shared between sweet corn and Napier fresh forage (Fig. 10). By contrast, for silage, only K00016 (l-lactate dehydrogenase) was shared in sweet corn and Napier. K00001 (alcohol dehydrogenase) and K00008 (l-iditol 2-dehydrogenase) were amongst the significantly enriched KOs in sweet corn silage. Heatmap of the functional profile was constructed using the differential abundance of KOs in both forage crops before and after ensiling (Fig. 11). The functional orthologs were primarily segregated by ensiling status, where the ensiled crops were clustered together and distinctly separated from the fresh forage crops. Supplementary Figure S7 shows that the metabolism of terpenoids and polyketides, glycan biosynthesis and metabolism and lipid metabolism were high in sweet corn and Napier silages. On the contrary, carbohydrate metabolism and nucleotide metabolism were enriched in sweet corn and Napier silages, respectively. Amino acid metabolism and metabolism of cofactors and vitamins decreased after ensiling in both crops. Figure 12 shows differentially abundant COG functional categories. Carbohydrate transport and metabolism, replication, recombination and repair, cell cycle control, cell division and chromosome partitioning, secondary metabolite biosynthesis, transport and catabolism were enriched after ensiling in both crops.

## Discussion

The fermentation properties of silage such as DM, pH, sugar content, microbial abundance and organic acid concentration in fresh crop and silage were determined (Table 1) to evaluate silage fermentation and quality. DM is a factor that can influence silage quality. The optimum DM content to produce good silage is between 30 and 40% DM^20^. In this study, the DM content of fresh crops was below 30% DM. This result may increase the possibility of spoilage. Spoilage is usually due to Clostridia fermentation^21^ caused by insufficient amounts of acids produced to inhibit fungal activity, which can initiate aerobic spoilage^16^. The spoilage of silage can be indicated by the production of butyric acid by more than 0.5% DM and high concentrations of propionic acid (0.3–0.5% DM) with high losses of DM and poor energy recovery^5,10^. However, this finding does not occur in this study as butyric acid and propionic acid were not detected in both silage samples (Table 1). Butyric and propionic acids are usually undetected in most good silages. Both Napier and sweet corn silage recorded pH values below 4.0; hence, this pH value may inhibit Clostridial growth. Clostridial fermentation can be prevented by promoting rapid proliferation of LAB which leads to decreased pH value^16^. Typically reported pH for corn and grass silages ranged from 3.7 to 4.0 and from 4.3 to 4.7, respectively^5^. Ensiling occurred when the epiphytic LABs convert the WSC into organic acids, particularly LA in anaerobic condition^22^ which reduces the pH. DM and CP decreased as oxygen was used by plant cells for respiration and proteolysis at the onset of ensiling^20^. However, the CP in sweet corn silage increased after ensiling in this study, which was also observed in other research^14^. This result might be due to excess proteins being produced by the fermentative microbial communities in S21. However, this result remains to be investigated by performing digestible protein (DCP) analysis and metabolic studies. WSC reduced after ensiling as observed in this study (Table 1) and other studies^23,24^. LA might contribute the most in reducing the silage pH as it is 10 to 12 times stronger than other organic acids such as acetic acid and propionic acid^5^. In the present study, the LA concentration in Napier and sweet corn significantly increased after 21 days of ensiling, which indicated that the LAB had initiated lactate fermentation in both forage crops. Studies by Yuan et al. and Li et al*.* showed that for tropical grasses such as Napier with low sugar availability, a prolonged ensiling period can shift the usual homofermentative LAB to heterofermentative LAB, generating acetic acid. This occurrence might be minimal in this study as the concentration of acetic acid was relatively low, which is below 1.0% DM, although the acetic acid content of Napier silage was relatively higher than that of sweet corn. The usual concentration of acetic acid in homofermentative ensiling ranges from 1 to 3% DM, and it is the second-highest organic acid detected after ensiling^5^. The moderate acetic acid concentration in silage may be beneficial because of its ability to inhibit yeast growth and activity (strong antifungal ability), thereby improving the aerobic stability of silage^16^. Collectively, the fermentation characteristics of silage produced in this study indicate successful silage fermentation. Apart from the physicochemical properties of silage, the sensory evaluation such as odour and colour of the silage can also provide a supplementary assessment but it was not conducted in this study.

Rarefaction curve analysis showed that the number of OTUs reached a plateau, implying that all unique OTUs have been sequenced in both forage crops. In this study, a significant increase or decrease in alpha diversity indexes indicated that bacterial communities underwent great changes after ensiling. Bacterial communities in fresh crops exhibited greater diversity than those in ensiled crops^25^, as observed in Napier where the bacterial diversity decreased after fermentation. Reduction of bacterial diversity was also observed in other studies for corn silage^12,13^ and Napier silage^26^. The decline of aerobic bacteria after ensiling is due to an anaerobic condition that is unfavourable for bacterial growth, and low pH contributes to the decrease of bacterial diversity. Conversely, bacterial diversity increased in sweet corn silage following ensiling. This result is also corroborated by Guan et al.^13^ and Xu et al.^15^ . We hypothesised that this result might be attributed to the inclusion of whole corn fruit in fermentation. Corn fruit contains high sugar content^27^ that can be utilised as the carbon source, causing the enrichment of various sugar-fermentable microbes to proliferate. In addition, the increment or reduction in alpha diversity following fermentation could be due to different source materials and climate^24^.

The aerobic microorganisms and facultative aerobes commonly dominate the pre-ensiling stage. LAB will increase and dominate after establishing the anaerobic condition. The abundance of *Lactobacillus* sp. of more than 70% in sweet corn and Napier silages has resulted in the decrease of species evenness and diversity with increased species dominance. Many studies reported that the predominance of *Lactobacillus* indicates high silage quality^12,28–31^. *Lactobacillus* produces LA and reduces the silage pH, thereby inhibiting undesirable spoilage bacteria. In fresh plant materials, the natural population of LAB is usually heterofermentative and low in number, however, it will begin to predominate, with more than 50% abundance^32,33^ in ensiled crops. In this study, *Lactobacillus* in fresh crops were only less than 5%, which is consistent with another study^29^. Bacterial communities in pre-ensiled crops are strongly correlated with the source material and the dominant taxa in the silage produced. In fresh Napier, the abundance of *Weissella* was 61.00%, which become the second dominant taxa after ensiling. The dominant genera in fresh sweet corn and Napier, namely, *Weissella, Pseudomonas, Leuconostoc* and *Pantoea,* were also predominant in other fresh crops such as soybean^34^ and *Moringa oleifera* leaves^35^*.* Major LAB, *Lactobacillus, Pediococcus, Lactococcus, Enterococcus, Streptococcus* and *Leuconostoce*^34,36^, which are commonly involved in ensiling of temperate forage crops, were also predominant in this study.

The co-occurrence network (Fig. 7) shows the interactions amongst bacterial taxa present in each silage. The taxa-taxa interaction in sweet corn was slightly more complex than that in Napier. Heterofermentative LAB, *Leuconostoc lactis* and *Leuconostoc mesenteroids*, which displayed strong positive correlations in sweet corn silage, indicate their cooperative relationships to populate the fresh crops. Both species are the common LABs dominating raw dairy cows’ milk, indicating possible horizontal transfer from fresh forage to milk during cow feeding, although the probability is small^37^. The consortia of heterofermentative and homofermentative LAB consisting of positively interacting *Lactobacillus brevis,* (heterofermentative) *Lactobacillus casei* (heterofermentative) and *Lactococcus lactis* (homofermentative) in sweet corn silage and positively interacting *Lactobacillus brevis, Lactobacillus casei* and *Lactobacillus fermentum* (heterofermentative) in Napier silage may indicate their synergistic action to produce LA and low level of acetic acid. This result could contribute to the decline of pH and growth inhibition of aerobic bacteria, leading to the success of silage fermentation. Therefore, we propose that these respective bacterial consortia should be explored as additives in fermenting sweet corn and Napier silages in tropical Malaysian climate. Moreover, in Napier, negative correlations were observed between *Lactobacillus brevis* and *Lactobacillus fermentum* with a plant pathogen, *Pseudomonas oryzahabitans* which could imply inhibitory activity between them. Furthermore, reduced proportions of several plant pathogens (*Pseudomonas oryzahabitans, Agrobacterium vitis, Xanthomonas sacchari)* and human pathogens (*Acinetobacter calcoaceticus* and *Chryseobacterium indologenes*) after ensiling suggest a plausible pathogen suppression effect.

High LA content in sweet corn and Napier silage is associated with the abundance of *Lactobacillus* and *Pediococcus*, where the principal metabolite of silage fermentation is LA^38^. Clostridia order was enriched in sweet corn silage, where it has a high correlation with LA content and low pH. Clostridium, an obligate anaerobe, can over-grow after LAB growth rate decreases in the silo, thereby affecting silage quality. It competes with LAB in the utilisation of sugar, amino acids or LAs as substrate^39^. The presence of *Clostridium* was unique in sweet corn silage; however, it does not cause spoilage as demonstrated in the fermentation characteristics (Table 1). Aerobic bacteria such as *Acetobacter*, *Klebsiella* and *Novosphingobium* were abundant in fresh forage and correlated with high DM, pH and WSC, which are usually the characteristics of fresh plants. LAB were dominant after ensiling as supported by the presence of high LA content in both silages. At the species level, positive correlations between heterofermentative and homofermentative species (Fig. 7) that dominated the ensiled crops (Supplementary Fig. S1) may indicate that the bacteria work synergistically to ferment sugars into LA and slight acetic acid. The high proportion of heterolactic *Lactobacillus fermentum* in the Napier silage (Supplementary Fig. S1) might explain the slightly higher acetic acid content in Napier than in sweet corn silage (Table 1).

The metagenomic functional prediction was conducted using Tax4Fun to predict the metabolic pathways involved during ensiling. The distinct ordination in the PCoA plot (Fig. 9) and heatmap (Fig. 11) indicated that the metabolisms differ greatly between silage and fresh forage crops. Carbon metabolism increased after ensiling in sweet corn (Supplementary Fig. S7a). Carbon metabolism is an essential pathway in ensiling because of the active utilisation of available sugar carbon by bacterial communities, particularly LAB^40^. During ensiling, the LAB will proliferate and utilise the available substrate to produce LA. High colonisation activity of LAB and overproduction of LA after ensiling may also justify the significantly higher energy metabolism and nucleotide metabolism observed in sweet corn and Napier silage. Fast energy turnover is necessary for active division of LAB cells. The decrease in amino acid metabolism after ensiling was also observed in another study^41^. In fresh forage crops, K00281 and K00163, which are responsible for amino acid metabolism, decreased after ensiling. This result might be due to the inhibition of amino acid metabolism from undesirable bacteria such as Clostridia. K00016 (l-lactate dehydrogenase) was more abundant after ensiling in sweet corn and Napier silage, which is related to organic acid production that might occur during the ensiling. Lactate dehydrogenase is an enzyme responsible for reversible conversion of pyruvate into lactate using NADH^42^. As oxygen is limited or unavailable once entering the anaerobic phase, it utilises the available glucose and produces LA. K00001 (alcohol dehydrogenase) and K00008 (l-iditol 2-dehydrogenase) are involved in alcohol production, where ethanol is commonly found in silages at low concentration^5^. Several microorganisms such as enterobacteria, yeasts and heterolactic acid bacteria can produce ethanol. There are few reviews of the literature that clearly link glycan biosynthesis and metabolism to fermentation. Enhancement of glycan biosynthesis and metabolism are also observed in other fermentation studies such as Indian idli batter^43^ and fermented pork fat^44^. Glycans are carbohydrate molecules commonly found on bacterial cell membranes that play certain roles in intrinsic or extrinsic recognition for cell–cell or microbe–microbe interaction^45^. We hypothesised that increased glycan biosynthesis and metabolism at day 21 could potentially indicate immense bacterial replication activity, and glycan provides a certain extent of stability and facilitates inter and intracellular communications amongst cells undergoing fermentation. Furthermore, increased metabolism of terpenoids and polyketides in sweet corn and Napier after ensiling might suggest an increase in the synthesis of terpenoids and polyketides that are often linked to antimicrobial activities for pathogen suppression^46^ (Fig. 7). Similarly, enrichment of COG functional categories related to bacterial division, carbohydrate transport and metabolism, and secondary metabolite production could imply vigorous utilisation of carbohydrates by actively multiplying fermentative bacteria to generate various metabolic end-products such as LA, acetic acid, terpenoids and polyketides. Silage bacteria utilise available substrates from plant cell walls such as hemicellulose and transport such substrates into, out or within a cell for bacterial cell division^17^. For example, heterofermentative LAB partially hydrolysed hemicellulose into pentoses and then finally into LA and acetic acid via the phosphoketolase pathway^17^.

Despite all possible discussions regarding the functions of bacterial communities in silage fermentation, the data obtained from functional prediction remain uncertain and should be examined with caution as it only relies on the 16S rRNA gene. The predicted functional profiles can be further validated through other omics studies such as transcriptomics, proteomics and metabolomics to provide a clear indication.

## Conclusion

The bacterial communities changed greatly after ensiling. The ensiled sweet corn and Napier were dominated by *Lactobacillus, Weissella*, *Pantoea, Pseudomonas* and *Leuconostoc.* LAB was predominant in both silages that exhibited a good range of fermentation properties, indicating successful ensiling. Hetero and homofermentative LAB species showed cooperative intra-relationship whilst showing negative correlations with bacterial pathogens, indicating synergistic fermentative action and pathogen suppression, respectively. Metabolic pathways related to fermentative activity of actively reproducing bacteria to ferment sugar carbons into organic acids, secondary metabolites and polyketides were enriched after ensiling. This study also proposed several heterofermentative and homofermentative indigenous LAB that could be used as additives to improve sweet corn and Napier silage fermentation under Malaysian climate. The information obtained in this study could provide comprehensive understanding of the bacterial communities associated with silage quality produced in Malaysia. The roles of important bacteria in fermentation could also be investigated using other advanced methods for future works.

## Methods

### Silage preparation

Fresh sweet corn and silage were collected at Padang Terap, Kedah (6.263313766240991, 100.58890571252691). The whole sweet corn plant (stalk, leaves, tassel and whole corn fruit) was harvested and chopped using a high-speed chopper. The plant materials were well mixed and then compacted into silo. Fresh Napier, including the stem, nodes and leaves, which were 40 days old, was harvested in Pondok Tanjung, Perak (5.031466358778387, 100.73149816957502) with 10 to 12 cm length from the soil and chopped using a high-speed chopper into 2 cm size. Permission was obtained from the owners of the private farm before collecting fresh crops and silage. The harvested fresh Napier was then ensiled in a laboratory silo in four replicates for 21 days (Supplementary Fig. S2). After ensiling, 400 g of silage was collected from the middle part of the silo and stored at − 20 °C for further analysis. All experimental protocols and plant handling involving sweet corn and Napier were conducted in accordance with the institutional and national guidelines.

### Fermentation analysis of naturally fermented silage

The fresh and ensiled crops were analysed for DM, WSC, LA content, CP and pH value. DM contents of fresh forage crops and silage were determined using the 934.01 AOAC method (2016) by drying in an oven (Etuve AC120; Froilabo, France) at 115 °C until the weight becomes constant. Water extracts of silage were obtained by homogenising the silage with distilled water at 10× dilution and filtering it with four layers of cheesecloth, and then the extract was centrifuged at 20,000×*g* at 4 °C for 20 min. The pH value of fresh crops and silage was recorded using a pH benchtop metre (STARA2110; ThermoScientific, England). The remaining water extract was kept at − 20 °C for further analysis. WSC was determined in accordance with the methods described by Dubois et al.^47^. The LA, acetic acid, propionic acid and butyric acid concentration was determined using HPLC (G1321A; Agilent Technologies, Germany). CP measurement was conducted using the Kjeldahl method, AOAC (2016).

### Bacterial enumeration

The microbial population was enumerated in fresh crops and silage. The water extracts of samples were prepared by homogenising 10 g of sample with 90 mL of sterile saline (0.85%) solution using an industrial blender (MX-GM1011; Panasonic, Malaysia) with 60 s interval. The mixture was filtered using a sterile Whatman filter paper (No. 40) and serially diluted. The culture medium used was MRS (CM361; Oxoid Ltd.) and nutrient agar (NA, CM1160; Oxoid Ltd.). NA plates were incubated at 30 °C for 24 h (aerobically) and MRS agar for 48 h in an anaerobic condition. Bacterial counts of LAB and aerobic bacteria were determined in log CFU/g of fresh materials as viable numbers of microorganisms.

### 16S rRNA gene amplicon sequencing

Ten grams of ensiled crops were mixed well with 50 mL of sterile saline (0.85%). The mixture was filtered, and extraction was conducted using the DNeasy Blood & Tissue Kit (Qiagen) as per the manufacturer’s protocol. The concentration of DNA was measured using a Nanodrop (ND-2000; Thermo Scientific, United States). The 16S rRNA gene (V3–V4) was amplified using the specific primers 341F (5′-CCTACGGGNGGCWGCAG-3′) and 805R (5′-GACTACHVGGGTATCTAATCC-3′) with sample-specific barcodes. Library preparation, pooling and sequencing were performed by Apical Scientific Sdn. Bhd. The libraries were normalised and pooled in accordance with the protocol recommended by Illumina and proceeded to next-generation sequencing using the MiSeq platform 300 PE.

### Sequence data analysis

Firstly, paired-end reads were subjected to removal of sequence adaptors and low-quality reads using BBDuk from the BBTools package (https://sourceforge.net/projects/bbmap/). The forward and reverse reads were merged using USEARCH v11.0.667 (https://www.drive5.com/usearch/). All sequences that were shorter than 150 bp or longer than 600 bp (sequenced on the MiSeq platform) were removed from downstream processing. Reads were then aligned with 16S rRNA (SILVA Release 132) and inspected for chimeric errors using VSEARCH v2.6.2. After quality assessment, reads were clustered de novo into OTUs at 97% similarity using UPARSE v11.0.667; rare OTUs with less than 2 reads (doubleton), which are often spurious, were deleted from downstream processing. Taxonomic assignment of OTU was achieved using QIIME V1.9.1 against the Silva 16S rRNA database (release 132). Sequence data were received as fastq files and submitted to the National Center for Biotechnology Sequence Read Archive under Bio Project accession number PRJNA497711.

### Statistical analyses

Microbial enumeration data were log-transformed prior to statistical analysis. Statistical analyses were performed using the Statistical Package for Social Sciences (SPSS Version 19.0, USA) to examine the differences amongst samples. Gabriel’s honest significant difference test was used for different sample means, and significance was declared at *p* < 0.05000. Bioinformatics analysis was performed using the MicrobiomeAnalyst web platform available at https://www.microbiomeanalyst.ca/^45^. The OTU table with taxa and metadata files were uploaded. Data were filtered using the following parameters: minimum count 4, 20% prevalence in samples of the low-count filter and 10% of the samples were removed in the low-variance filter. Data scaling was conducted using total sum scaling, with no data transformation and data rarefying. In alpha diversity, significant differences amongst different groups were calculated on the basis of the ANOVA/t-test at *p-*value < 0.05. Beta diversity was calculated on the basis of the Bray–Curtis distance, and statistical comparisons amongst groups were performed using PERMANOVA. LEfSe analysis was conducted at feature level with LDA score > 4.0 capped at FDR-adjusted *p*-value < 0.05. Heatmap was constructed on the basis of the OTU observed in LEfSE analysis (Fig. 6). The clusters were separated by crop type and by fermentation status. Co-occurrence network analyses were conducted to evaluate the complexity of bacterial interactions in fresh forage and silage. The correlations were calculated using SparCC analysis with correlation value of > 0.6 and 200 permutations with *p*-value < 0.05000 (Fig. 7). CCA analysis of significant fermentation properties data and differentially abundant taxa were constructed on the basis of each crop. Venn diagram was constructed using a web interface tool, namely, InteractiVenn^47^. Functional profiles were determined by analysing the OTUs using Tax4Fun in MicrobiomeAnalyst.

## Supplementary Information

Supplementary Information.

## Data Availability

The raw sequence reads were deposited into the NCBI Sequence Read Archive (SRA; http://www.ncbi.nlm.nih.gov/Traces/sra/) database under the Accession Numbers SRR12972050 to SRR12972077.
