# Supplementary material for: Bacterial communities associated with silage of different forage crops in Malaysian climate analysed using 16S amplicon metagenomics
Source: Sci Rep. 2022 May 2;12:7107. doi: 10.1038/s41598-022-08819-4 (PMC9061801; doi:10.1038/s41598-022-08819-4)
Supplement: Supplementary file 1 — Supplementary Information. [file 41598_2022_8819_MOESM1_ESM.pdf]

Supplementary Information

Bacterial Communities Associated with Silage of Different Forage Crops in Malaysian Climate Analysed Using 16S Amplicon Metagenomics

Minhalina Badrul Hisham[1, 2], Amalia Mohd Hashim[2,6*], Nursyuhaida Mohd Hanafi[1*], Norafizah Abdul Rahman[1], Nur Elina Abdul Mutalib[3ǂ], Chun Keat Tan[1ǂ], Muhamad Hazim Nazli[4] and Nur Fatihah Mohd Yusoff[5]

[1] Agro-Biotechnology Malaysia Institutes (ABI), National Institutes of Biotechnology Malaysia (NIBM), Ministry of Science, Technology and Innovation (MOSTI) c/o MARDI Headquarters, Serdang, 43400, Selangor, Malaysia.

[2] Department of Microbiology, Faculty of Biotechnology and Biomolecular Sciences, Universiti Putra Malaysia, 43400, Serdang, Selangor, Malaysia.

[3] Institutes for Health Systems Research, National Institutes of Health Malaysia (NIH), 40170 Shah Alam, Selangor, Malaysia.

[4] Department of Crop Science, Faculty of Agriculture, Universiti Putra Malaysia, 43400 Serdang, Selangor, Malaysia

[5] Department of Cell and Molecular Biology, Faculty of Biotechnology and Biomolecular Sciences, Universiti Putra Malaysia, 43400 Serdang, Selangor, Malaysia

[6] Halal Products Research Institute, Universiti Putra Malaysia, 43400 Serdang, Selangor, Malaysia

* Corresponding Author, nursyuhaida@nibm.my* & amalia@upm.edu.my *
ǂthese authors contributed equally to this work

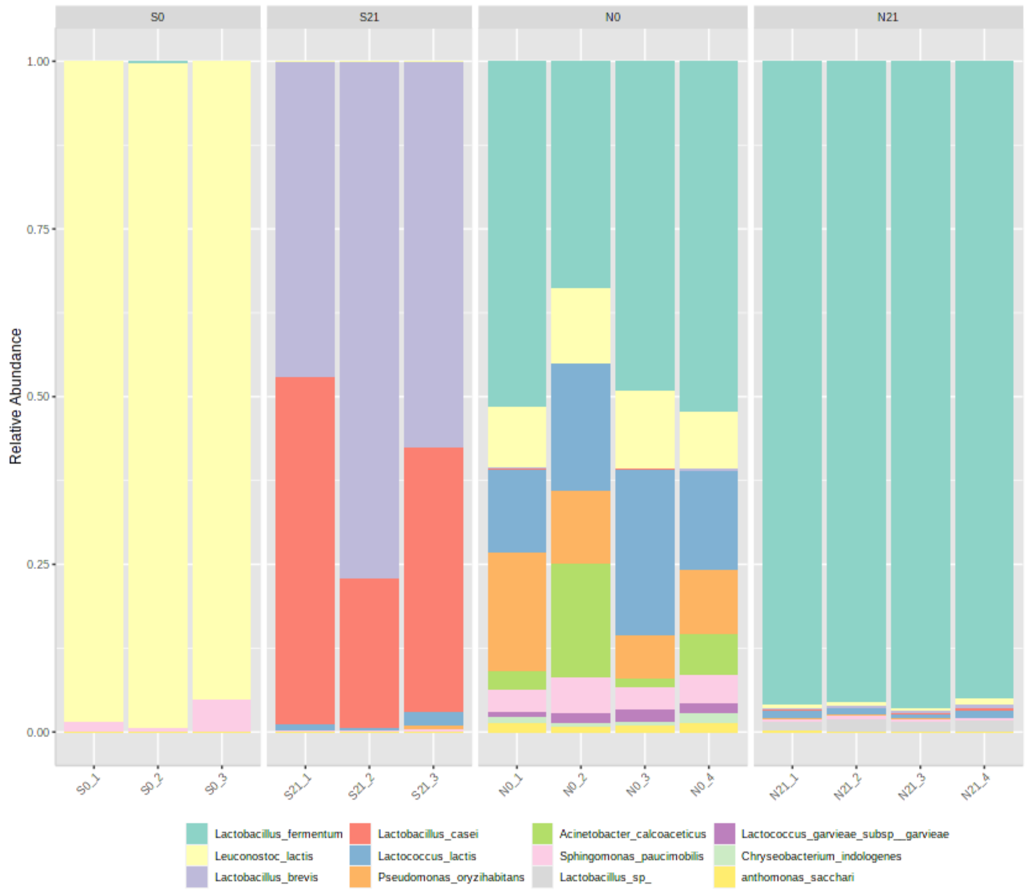

Figure S1. Composition of bacterial communities before and after ensiling using unrarefied sequences at species level for each sample. Top 12 species are shown. Unassigned species are not shown.

(a)

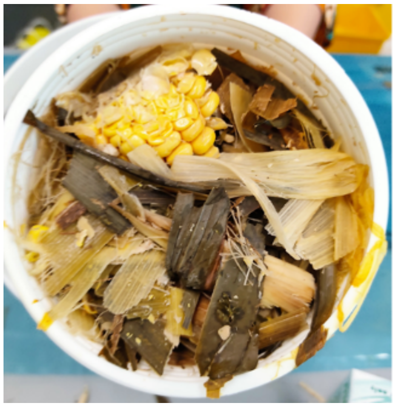

(b)

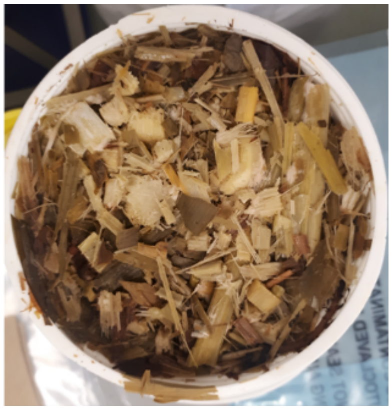

Figure S2. Silage in a laboratory-size silo after 21 days of fermentation: **(a)** sweet corn and **(b)** Napier

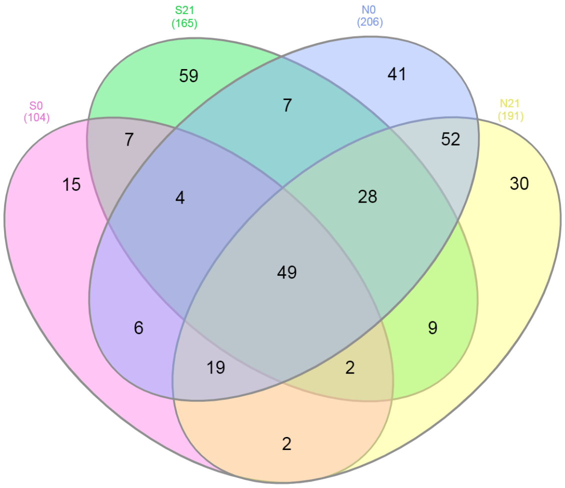

Figure S3 Venn diagram of unique and common OTUs in fresh forage and silage for sweet corn and Napier (S0, Fresh sweet corn; S21, Sweet corn silage; N0, Fresh Napier; N21, Napier silage)

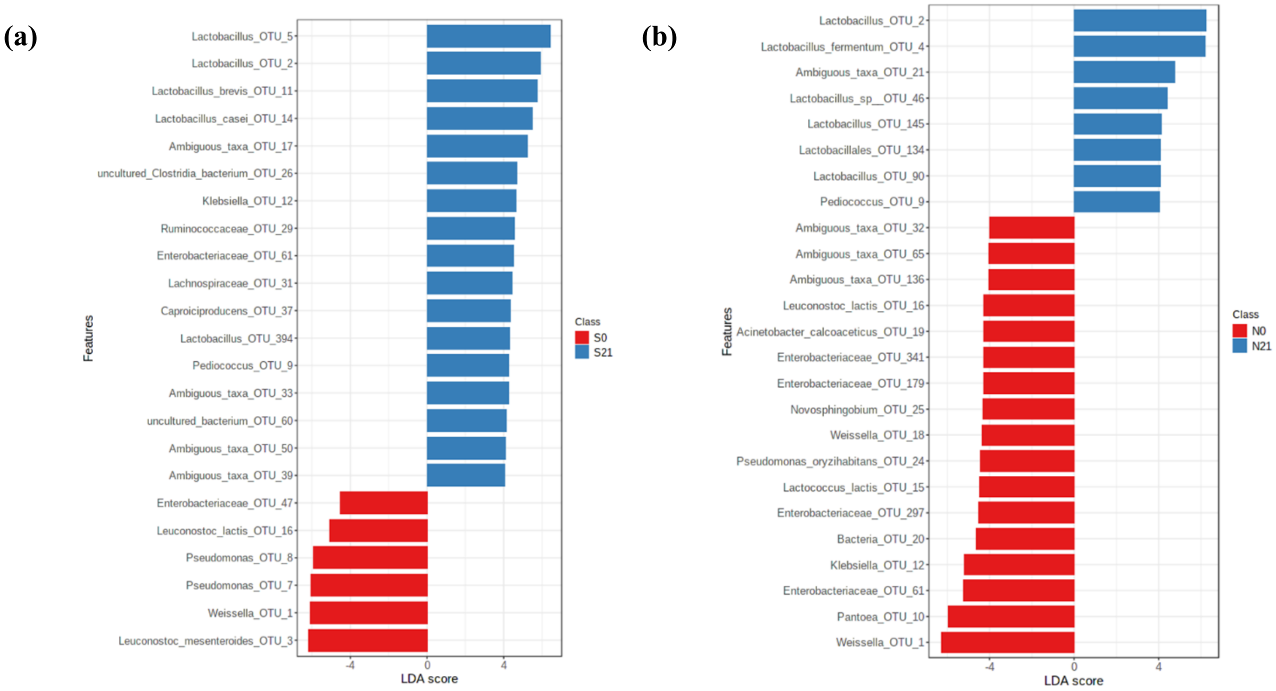

Figure S4 Graphics of linear discriminant analysis (LDA) effect size (LEfSe) between fresh forage and silage: **(a)** sweet corn and **(b)** sweet corn at feature level. Red indicates fresh forage and blue for silage. The threshold on the logarithmic LDA score for discriminative features was set to 4.0 at *p*-value < 0.05 (FDR-adjusted). (S0, Fresh sweet corn; S21, Sweet corn silage; N0, Fresh Napier; N21, Napier silage).

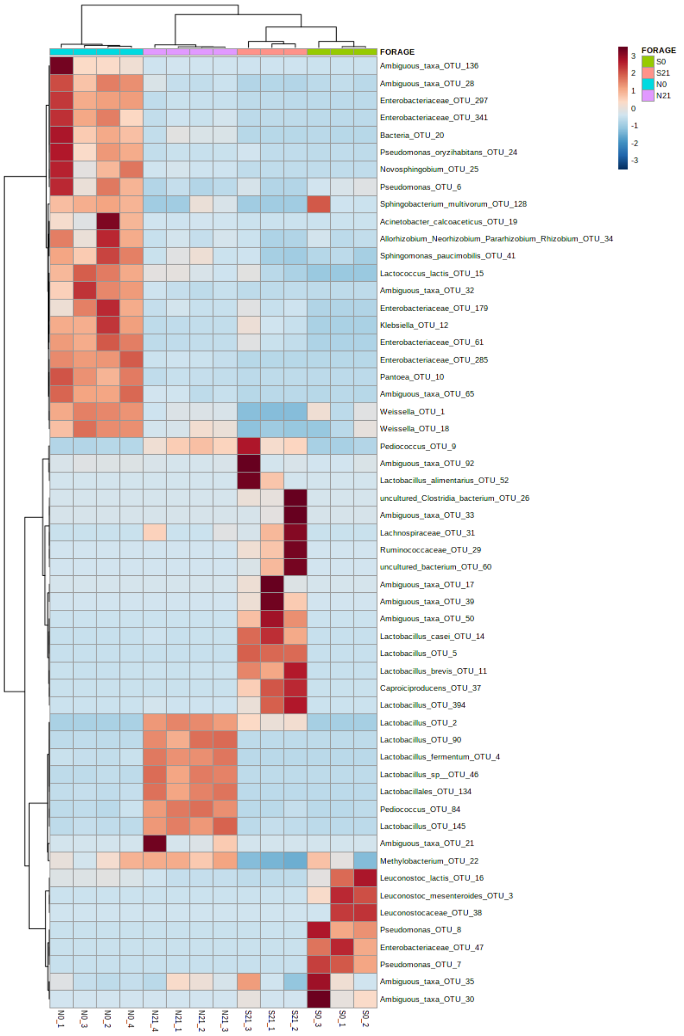

Figure S5 Heatmap constructed using differentially abundant OTUs based on LEFSe analysis at feature level. Pearson distance was used with the Ward clustering algorithm. Red indicates an increased abundance, whereas blue indicates a decreased abundance. The darker the color, the higher the abundance. (S0, Fresh sweet corn; S21, Sweet corn silage; N0, Fresh Napier; N21, Napier silage).

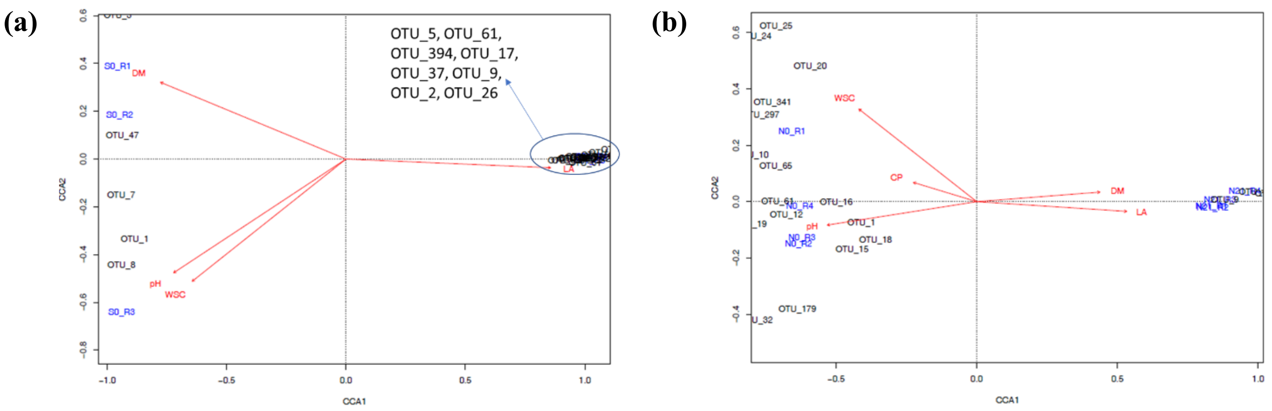

Figure S6 Correlation between physicochemical properties and bacterial taxa of fresh forage and silage using CCA analysis: **(a)** sweet corn and **(b)** Napier. (OTU_3, *Leuconostoc mesenteroides*; OTU_7, OTU_8, *Pseudomonas*; OTU_9, *Pediococcus*; OTU_10, *Pantoea*; OTU_12, *Klebsiella*; OTU_15, *Lactococcus lactis*; OTU_17, *Rummeliibacillus*; OTU_20, *Bacteria*; OTU_22, *Methylobacterium*; OTU_25, *Novosphingobium*; OTU_28, *Curtobacterium*; OTU_32, *Lactococcus*; OTU_35, *Aureimonas*; OTU_38, *Leuconostocaceae*; OTU_43, *Cellulomonas*; OTU_61, OTU_179, OTU_297, *Enterobacteriaceae*; OTU_65, *Kosakonia*; OTU_84, *Pediococcus*; OTU_91, *Exiguobacterium*)

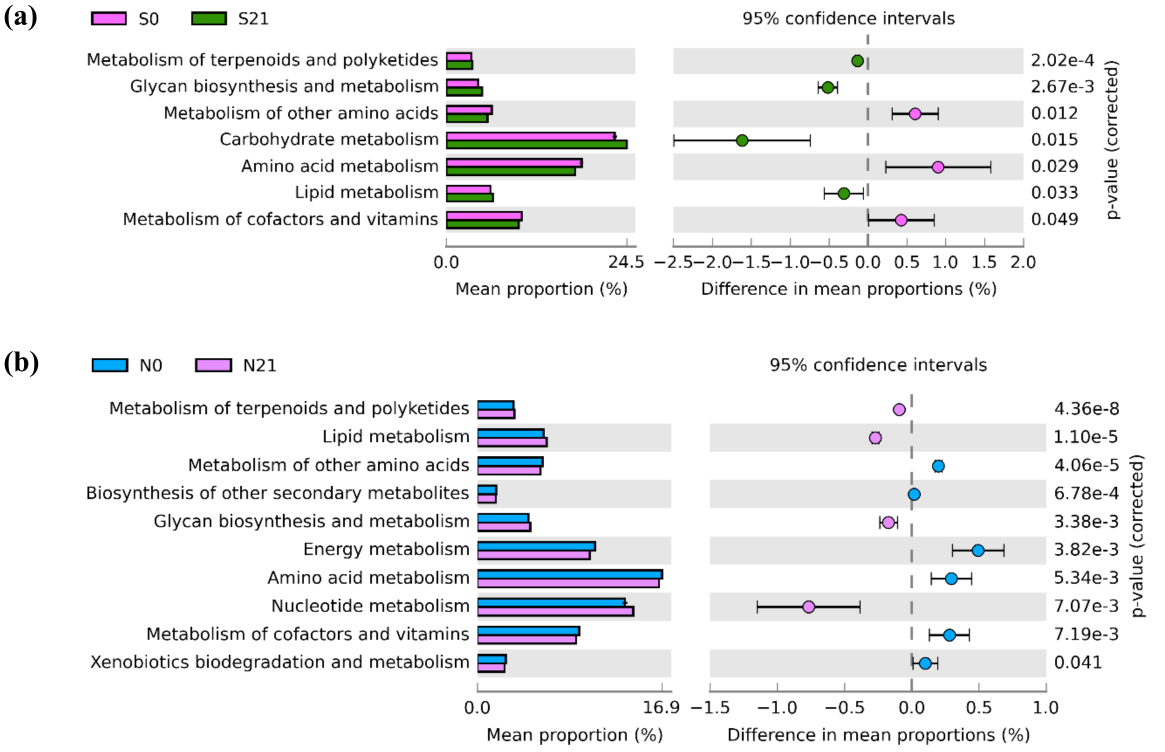

Figure S7 Significantly different KEGG metabolisms before and after ensiling using STAMP analysis: **(a)** sweet corn and **(b)** Napier

Table S1 Read counts, number of singletons, number of sequences and Goods coverage for fresh forage and silage samples.

Sample	Read counts	No. of singleton	No. of seqs	Goods coverage
**S0_1**	63825	8	63617	99.98742
**S0_2**	66073	11	65863	99.98330
**S0_3**	57979	7	57910	99.98791
**S21_1**	45328	14	39521	99.96458
**S21_2**	67193	12	61440	99.98047
**S21_3**	66047	4	62818	99.99363
**N0_1**	59943	4	59753	99.99331
**N0_2**	33172	6	33089	99.98187
**N0_3**	69834	6	69715	99.99139
**N0_4**	55339	4	55165	99.99275
**N21_1**	39633	12	39565	99.96967
**N21_2**	33161	15	33089	99.95467
**N21_3**	49048	9	48914	99.98160
**N21_4**	35723	14	35563	99.96063

Table S2 Percentage abundance of bacterial communities at the genus level.

	N0	N21	S0	S21
Weissella	61.00	23.84	25.43	1.36
Pantoea	19.65	0.81	0.01	0.06
Not Assigned	7.05	1.26	0.83	1.67
Lactobacillus	3.50	70.78	0.04	93.78
Klebsiella	3.25	0.43	0.01	0.99
Lactococcus	1.20	0.33	0.00	0.23
Pseudomonas	0.63	0.04	40.89	0.19
Novosphingobium	0.54	0.08	0.00	0.00
Leuconostoc	0.53	0.17	32.49	0.10
Methylobacterium	0.39	0.43	0.17	0.04
Acinetobacter	0.35	0.01	0.00	0.00
Sphingomonas	0.25	0.09	0.04	0.04
Cronobacter	0.24	0.01	0.00	0.01
Kosakonia	0.24	0.02	0.00	0.00
Allorhizobium Neorhizobium Pararhizobium Rhizobium	0.17	0.03	0.02	0.02
Curtobacterium	0.14	0.02	0.01	0.00
Chryseobacterium	0.12	0.01	0.00	0.00
Escherichia Shigella	0.10	0.01	0.00	0.03
Acidisoma	0.10	0.02	0.00	0.00
Acidovorax	0.07	0.01	0.00	0.00
Xanthomonas	0.06	0.01	0.00	0.00
Rothia	0.05	0.00	0.00	0.01
Kocuria	0.05	0.05	0.00	0.01
Stenotrophomonas	0.05	0.01	0.01	0.00
Herbaspirillum	0.04	0.01	0.00	0.00
Mucilaginibacter	0.03	0.00	0.00	0.00
Pediococcus	0.03	0.33	0.01	0.43
X1174_901_12	0.03	0.03	0.00	0.00
Pseudoxanthomonas	0.03	0.01	0.00	0.00
Massilia	0.02	0.00	0.00	0.00
Burkholderia Caballeronia Paraburkholderia	0.02	0.00	0.01	0.09
Aureimonas	0.01	0.02	0.03	0.01
Siphonobacter	0.01	0.00	0.00	0.00
Bosea	0.01	0.00	0.00	0.00
Acetobacter	0.00	1.13	0.00	0.00
Anaerocolumna	0.00	0.01	0.00	0.10
Caproiciproducens	0.00	0.00	0.00	0.47
Lachnotalea	0.00	0.00	0.00	0.32
Ruminiclostridium	0.00	0.00	0.00	0.04

Table S3 Taxa-taxa correlation in sweet corn silage using SPARCC set at permutation value of 200, *p-value* < 0.05, correlation threshold of >0.9.

Taxon1	Taxon2	Correlation	*p-value*
Leuconostoc mesenteroides	*Leuconostoc lactis*	0.9946	0.02490
uncultured bacterium	*Lactococcus lactis*	0.9420	0.02490
Lactobacillus casei	*Lactobacillus brevis*	0.9350	0.04480
Lactococcus lactis	*Lactobacillus casei*	0.9301	0.03980
uncultured bacterium	*Lactobacillus casei*	0.9181	0.02980
uncultured bacterium	*Lactobacillus brevis*	0.9100	0.02490
Lactococcus lactis	*Lactobacillus brevis*	0.9097	0.03980

Table S4 Taxa-taxa correlation in Napier silage using SPARCC set at permutation value of 200, *p-value* < 0.05, correlation threshold of >0.6.

Taxon1	Taxon2	Correlation	*p-value*
Lactobacillus fermentum	*Leuconostoc lactis*	1	0.04480
Lactobacillus brevis	*Lactobacillus fermentum*	0.9921	0.01000
Lactobacillus casei	*Lactobacillus sp*	0.9370	0.01000
Lactococcus garvieae subsp garvieae	*Xanthomonas sacchari*	0.9172	0.02490
Pseudomonas oryzihabitans	*Xanthomonas sacchari*	0.9134	0.01990
Lactococcus garvieae subsp garvieae	*Pseudomonas oryzihabitans*	0.9129	0.01990
Chryseobacterium indologenes	*Xanthomonas sacchari*	0.9085	0.02490
Agrobacterium vitis pv musae	*Pseudomonas oryzihabitans*	0.9083	0.03480
Acinetobacter calcoaceticus	*Agrobacterium vitis pv musae*	0.9006	0.02490
Acinetobacter calcoaceticus	*Pseudomonas oryzihabitans*	0.8947	0.01490
Lactobacillus fermentum	*Lactobacillus sp*	0.8773	0.01990
Lactobacillus brevis	*Lactobacillus sp*	0.8593	0.01490
Agrobacterium vitis pv musae	*Chryseobacterium indologenes*	0.8522	0.04980
Acinetobacter calcoaceticus	*Lactococcus garvieae subsp garvieae*	0.8310	0.02490
Lactobacillus casei	*Lactobacillus fermentum*	0.6940	0.04980
Lactobacillus casei	*Lactobacillus brevis*	0.6742	0.01000
Acinetobacter calcoaceticus	*Leuconostoc lactis*	-1	0.04980
Leuconostoc lactis	*Acinetobacter calcoaceticus*	-1	0.04980
Leuconostoc lactis	*Pseudomonas oryzihabitans*	-1	0.04480

Table S5 KEGG Functional Orthologs (KOs) and its definition based on KEGG Orthology Database.

KO Number	Definition
**K00001**	alcohol dehydrogenase
**K00008**	L-iditol 2-dehydrogenase
**K00013**	histidinol dehydrogenase
**K00016**	L-lactate dehydrogenase
**K00020**	3-hydroxyisobutyrate dehydrogenase
**K00029**	malate dehydrogenase
**K00040**	fructuronate reductase
**K00058**	D-3-phosphoglycerate dehydrogenase / 2-oxoglutarate reductase
**K00065**	2-dehydro-3-deoxy-D-gluconate 5-dehydrogenase
**K00097**	4-hydroxythreonine-4-phosphate dehydrogenase
**K00100**	butanol dehydrogenase
**K00105**	alpha-glycerophosphate oxidase
**K00123**	formate dehydrogenase major subunit
**K00131**	glyceraldehyde-3-phosphate dehydrogenase (NADP+)
**K00135**	succinate-semialdehyde dehydrogenase
**K00145**	N-acetyl-gamma-glutamyl-phosphate reductase
**K00158**	pyruvate oxidase
**K00163**	pyruvate dehydrogenase E1 component
**K00164**	2-oxoglutarate dehydrogenase E1 component
**K00219**	2,4-dienoyl-CoA reductase (NADPH2)
**K00231**	protoporphyrinogen/coproporphyrinogen III oxidase
**K00244**	fumarate reductase flavoprotein subunit
**K00249**	acyl-CoA dehydrogenase
**K00257**	acyl-ACP dehydrogenase
**K00259**	alanine dehydrogenase
**K00262**	glutamate dehydrogenase (NADP+)
**K00265**	glutamate synthase (NADPH) large chain
**K00281**	glycine dehydrogenase
**K00335**	NADH-quinone oxidoreductase subunit F
**K00336**	NADH-quinone oxidoreductase subunit G
**K00341**	NADH-quinone oxidoreductase subunit L
**K00342**	NADH-quinone oxidoreductase subunit M
**K00343**	NADH-quinone oxidoreductase subunit N
**K00344**	NADPH:quinone reductase
**K00359**	NADPH:quinone reductase
**K00364**	GMP reductase
**K00381**	sulfite reductase (NADPH) hemoprotein beta-component
**K00382**	dihydrolipoamide dehydrogenase
**K00383**	glutathione reductase (NADPH)

**K00384**	thioredoxin reductase (NADPH)
**K00527**	ribonucleoside-triphosphate reductase (thioredoxin)
**K00548**	5-methyltetrahydrofolate--homocysteine methyltransferase
**K00599**	tRNA N(3)-methylcytidine methyltransferase METTL6
**K00680**	uncharacterized N-acetyltransferase
**K00689**	dextransucrase
**K00694**	cellulose synthase (UDP-forming)
**K00754**	L-malate glycosyltransferase
**K00756**	pyrimidine-nucleoside phosphorylase
**K00763**	nicotinate phosphoribosyltransferase
**K00790**	UDP-N-acetylglucosamine 1-carboxyvinyltransferase
**K01390**	IgA-specific metalloendopeptidase
**K08643**	zinc metalloprotease ZmpB
